# Features of spatial and functional segregation and integration of the primate connectome revealed by trade-off between wiring cost and efficiency

**DOI:** 10.1371/journal.pcbi.1005776

**Published:** 2017-09-29

**Authors:** Yuhan Chen, Shengjun Wang, Claus C. Hilgetag, Changsong Zhou

**Affiliations:** 1 National Key Laboratory of Cognitive Neuroscience and Learning, Beijing Normal University, Beijing, China; 2 Beijing Key Laboratory of Brain Imaging and Connectomics, Beijing Normal University, Beijing, China; 3 IDG/McGovern Institute for Brain Research, Beijing Normal University, Beijing, China; 4 Department of Physics, Hong Kong Baptist University, Kowloon Tong, Hong Kong; 5 Centre for Nonlinear Studies, and Beijing-Hong Kong-Singapore Joint Centre for Nonlinear and Complex Systems (Hong Kong), Institute of Computational and Theoretical Studies, Hong Kong Baptist University, Kowloon Tong, Hong Kong; 6 Department of Physics, Shaanxi Normal University, Xi’An, Shaanxi Province, China; 7 Institute of Computational Neuroscience, University Medical Center Eppendorf, Hamburg, Germany; 8 Department of Health Sciences, Boston University, Boston, Massachusetts, United States of America; 9 Beijing Computational Science Research Center, Beijing, China; 10 Research Centre, HKBU Institute of Research and Continuing Education, Shenzhen, China; Max Planck Institute of Neurobiology, GERMANY

## Abstract

The primate connectome, possessing a characteristic global topology and specific regional connectivity profiles, is well organized to support both segregated and integrated brain function. However, the organization mechanisms shaping the characteristic connectivity and its relationship to functional requirements remain unclear. The primate brain connectome is shaped by metabolic economy as well as functional values. Here, we explored the influence of two competing factors and additional advanced functional requirements on the primate connectome employing an optimal trade-off model between neural wiring cost and the representative functional requirement of processing efficiency. Moreover, we compared this model with a generative model combining spatial distance and topological similarity, with the objective of statistically reproducing multiple topological features of the network. The primate connectome indeed displays a cost-efficiency trade-off and that up to 67% of the connections were recovered by optimal combination of the two basic factors of wiring economy and processing efficiency, clearly higher than the proportion of connections (56%) explained by the generative model. While not explicitly aimed for, the trade-off model captured several key topological features of the real connectome as the generative model, yet better explained the connectivity of most regions. The majority of the remaining 33% of connections unexplained by the best trade-off model were long-distance links, which are concentrated on few cortical areas, termed long-distance connectors (LDCs). The LDCs are mainly non-hubs, but form a densely connected group overlapping on spatially segregated functional modalities. LDCs are crucial for both functional segregation and integration across different scales. These organization features revealed by the optimization analysis provide evidence that the demands of advanced functional segregation and integration among spatially distributed regions may play a significant role in shaping the cortical connectome, in addition to the basic cost-efficiency trade-off. These findings also shed light on inherent vulnerabilities of brain networks in diseases.

## Author summary

The intricate primate structural connectome, as the network substrate for distributed and integrated brain function, is shaped by fundamental physical factors and functional requirements. We addressed a trade-off between two competing basic factors of wiring cost and processing efficiency as well as additional requirements of functional segregation and integration on regional structural connectivity profiles by applying cost–efficiency trade-off model to reconstruct the *macaque* cortical network. We also compared this model with a generative model combining spatial distance and topological similarity. The trade-off model balancing the two basic factors recovered the main topological features that were explicitly specified as the objective functions in the generative model. Moreover, 67% of all connections and most regional connectivity profiles were recovered by the cost-efficiency trade-off, substantially outperforming the generative model. Most long-distance links, unexplained by the cost-efficiency trade-off, are concentrated on a few special regions constituting long-distance connectors (LDCs). Departing from previous findings, LDCs are mostly non-hubs, but are crucial for supporting functional integration and, counter-intuitively also for proper segregation across different hierarchical scales of the cortical network. The perspective of a trade-off between basic factors on brain structural organization sheds light on the organization principles and potential vulnerabilities of the cortical connectome.

## Introduction

The large-scale network of cortical areas in the brain supports and integrates brain functions that are distributed across spatially segregated regions [1]. It has been known for a long time that different regions have specialized roles in brain function [2, 3] and such segregated functions also need to be integrated, a finding supported by neuroimaging studies [4–12].

The primate brain connectome is a physical network with nodes (cortical regions) and connections (fiber projections) embedded in Euclidean space [13, 14]. Exploring the factors that shape the spatial layout and the topological organization of the connectome is important for understanding the organization principles and structure-function relationships in the brain that support both segregated and integrated functioning at the systems level. It has been generally believed that the brain connectome reflects a trade-off between physical cost and functional requirements [13]. Early work mainly focused on testing the hypothesis of wiring cost minimization using component placement optimization [15–17], by fixing the topological connectivity of the network. While the placement of regions in some sub-systems (e.g., *macaque* monkey frontal cortex and *C*. *elegans* ganglia) appeared to be optimal [15–18], the global network appears not to minimize wiring cost, and comprises a substantial admixture of long-distance connections [13]. A scheme of wiring cost minimization with fixed network topology does not permit to explicitly study the competition between physical cost and network functionality. However, it is also an unresolved question of how to quantify the functional requirements of the brain. In such a spatially embedded signal communication brain network, the interregional connection patterns may need to achieve a balance between reducing cost by sending most projections to spatially close nodes while still maintaining efficient communication among spatially distant components, which may be a possible function of some of the long-distance connections [14, 18]. The processing efficiency of a network, as measured by the inverse of shortest graph path-length, thus, may be employed to partially capture the network functionality for a quantitative study of the trade-off with the wiring cost minimization. This approach is indeed reasonable, since it has been found that the processing efficiency of the neural network is related to cognitive performance and dysfunction for brain disorders and diseases [19–21].

Recently, a great amount of attention has been given to characterizing important features of the organization of neural networks [14, 22–27] and to exploring possible basic factors or simple rules which may play a role in generating such features. Graph theoretical approaches have revealed characteristic topological features of the cortical connectome, such as densely connected modules [28, 29], hubs with a large number of connections [30, 31], and rich-clubs with dense connections among hubs [32]. Our recent work considered a trade-off between wiring cost and processing efficiency by fixing the spatial layout of the cortical areas [27] and showed that prominent network properties, such as module divisions and spatial positions of the hubs may be determined by such a trade-off [27]. Generative models combining observed spatial features (e.g., connectivity decaying with distance) and topological features (e.g., matching index measuring similar input and output patterns of nodes) [14, 22–26] can reproduce some other statistical properties of the real connectivity, such as the distribution of degree, clustering, betweenness centrality and edge length. However, as shown in S1 Fig, it is still challenging to reproduce the degree sequence of individual cortical nodes by the generative model. The heterogeneous degrees may be also affected by the cost-efficiency trade-off, as the degrees from the cost-efficiency optimized model are significantly correlated with the real degrees in different nervous systems (*macaque* and *C*. *elegans*) [27]. More importantly, the different spatial and topological features of brain networks may not be independent, but rather be essentially related to basic physical constraints (e.g., wiring cost minimization) [22]. For instance, the topological similarity (in terms of common neighbors) of different regions is also affected by the wiring cost [25]. Although the important statistical features in the neural networks may be captured by the combination of a few observed factors [13, 14, 33, 34], a fundamental but still not well understood question is how the regional connectivity profiles as well as individual connections are shaped by some basic principles, for instance, wiring economy in trade-off with efficient processing. Which model can better capture the rules that shape the regional connectivity and its relationship to functions, a generative model combining different observed features or a trade-off model with basic factors of the wiring cost and processing efficiency? Is the arrangement of wiring-costly long-distance connections among different regions related to the segregation and integration of diverse brain functions? Exploring how such basic rules shape the cortical connectivity and its relationship to advanced functional requirements deepens the understanding of the relationships among the underlying design principles, wiring diagrams, functional performance, and potential vulnerability in the primate brain connectome.

Here we studied how the trade-off between two basic factors, the wiring cost and the topological processing efficiency, affects other topological features and regional connectivity profiles of the primate cortex structural connectome, and explored the relationship between the long-distance connections and functional requirements of segregation and integration. Previously, it was found that the degree distribution may be related to different factors, such as the cost-efficiency trade-off [27], or the combination of wiring distance and matching index [14] (S1 Fig). Though it is significant, the relatively low correlation between the degrees in the real network and that from the cost-efficiency trade-off model (without fixing degrees) implies that the degree sequence of individual nodes is also shaped by other factors. Indeed, even though the generative model [14] can closely recover the degree distribution, it cannot reliably reproduce the degree sequence of the nodes (S1 Fig). Therefore, in this study, the input and output degrees of the nodes are fixed as a constraint together with the spatial layout in the cost-efficiency trade-off model to obtain reconstructed networks from the combined optimization of wiring cost and processing efficiency. For comparison, we also consider the objective function of a generative model [14, 24] explicitly aimed at reproducing multiple statistical features of the connectome. We extended the generative model to consider the constraint of fixed degree as in the real network for a fair comparison with the trade-off model. We searched the best generative model with the most similar distribution of several features compared to the real *macaque* cortical network, and compared the performance of the optimized networks from the models in the recovery of topological features, regional connectivity profiles and individual connections. Our analyses showed that the trade-off outperforms the generative model in these aspects. The detailed comparison of real connectivity with the trade-off model for each region allowed us to determine (1) how much connectivity of the whole network and each region can be explained by the cost-efficiency trade-off, (2) how the unexplained connections are distributed among different functional regions and domains, and (3) what relationships exist between regions possessing many long-distance connections and the overarching functional features of segregation and integration. Our analyses provide evidence that the wiring cost-efficiency trade-off plays an essential role in determining regional cortical connectivity. However, additional requirements for proper segregation and integration of functions can strongly violate the cost minimization for some cortical regions and induce many long-range connections. In contrast to typical hubs identified by previous studies, these regions are typically non-hubs, but concentrate the long-distance projections in the network, acting as long-distance connectors (LDCs). While the organization of LDCs could reduce the metabolic and functional burden of outstanding hubs, such regions may also become spots of vulnerability in the primate brain network. Overall, the results provide support for, and insights into, the trade-off between physical cost and functional values of the primate brain connectome organization.

## Results

### The primate cortical network displays a cost-efficiency trade-off

**Fig 1:**
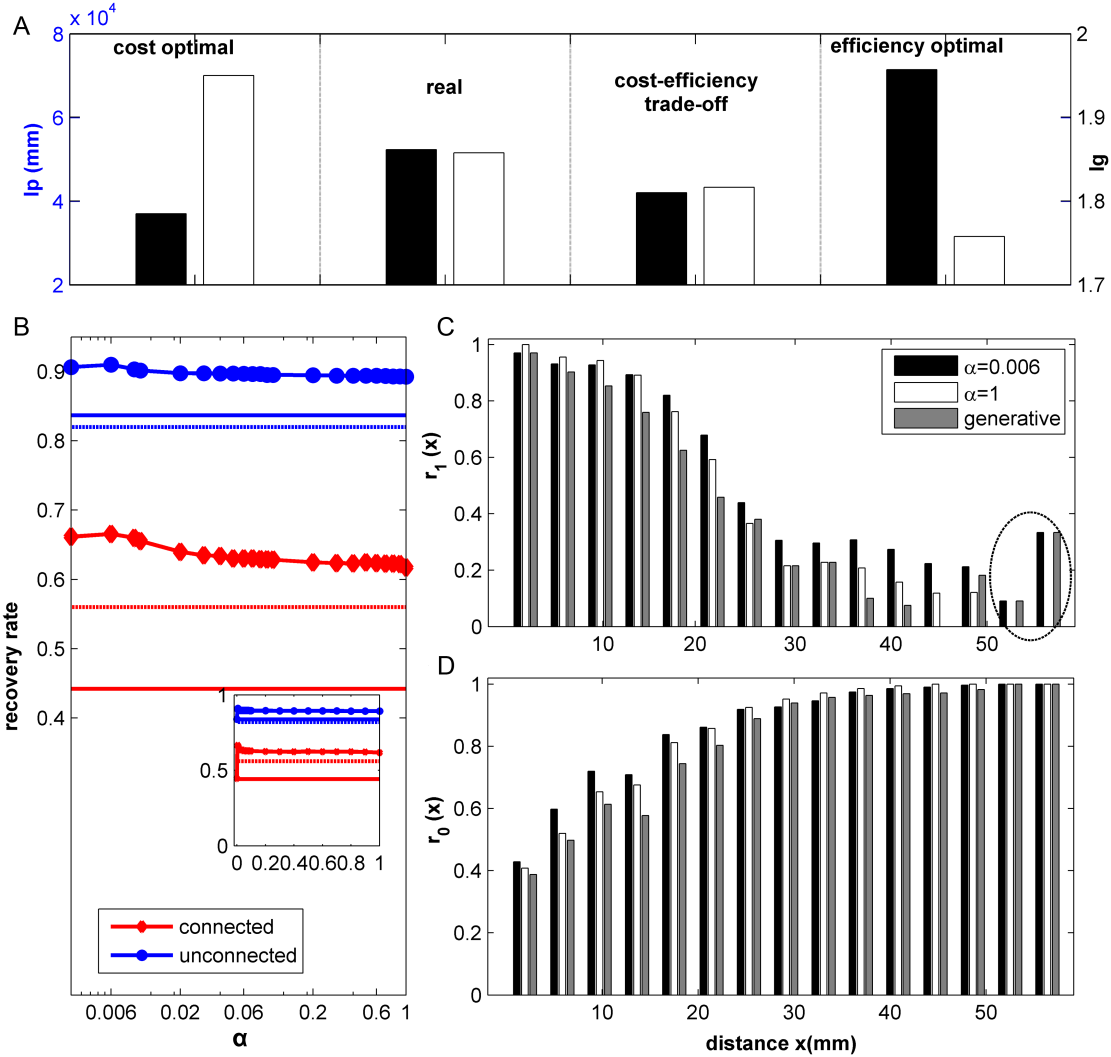
Cost-efficiency trade-off in the primate cortical connectome and comparison to the generative model. (A) Wiring cost *l*_*p*_ (black bar) and graph path length *l*_*g*_ (white bar) in the real network and reconstructed networks with optimal wiring cost or efficiency, respectively, or their trade-off that maximally recovers the real connectivity. (B) Recovery rates *r*_1_ (red) for all connected pairs and *r*_0_ (blue) for all unconnected pairs in the real network, as functions of α (log scale). Inset: α on linear scale. The solid line with symbol represents the recovery rate by the trade-off model. The solid line without symbols denotes the corresponding results for random benchmarks. The dashed red or blue lines show the corresponding results by the extended generative model with fixed degrees. (C) and (D): *r*_1_ and *r*_0_ as a function of the spatial distance *x* between the pairs of areas in the real network, for α = 0.006 (black bars), α = 1 (white bars) in the cost-efficiency trade-off model, and in the extended generative model with fixed degrees (grey bars).

Fig 1A shows that the real network indeed displays a trade-off between wiring cost and processing efficiency. In this work, the cost-efficiency model refers to the case with fixed degrees as in the real network. Optimizing wiring cost on its own leads to a physically cheaper network, which is, however, less efficient; with the wiring cost *l*_*p*_ reduced to 77.1% and the efficiency *l*_*g*_ increased to 105% of the real network. By contrast, optimizing the processing efficiency on its own increases efficiency (*l*_*g*_ reduced to 94.7%), but at the expense of wiring cost (*l*_*p*_ increased to 113.7%). The model combining wiring cost and efficiency brings both *l*_*p*_ and *l*_*g*_ closer to the real network, as seen by the configuration that maximally recovers the real connectivity.

The rates of link recovery with respect to α in Fig 1B–1D clearly show that a trade-off between wiring cost and processing efficiency can give a better account of the real connectivity than the individual factors. We computed the recovery rate *r*_1_ for the connected pairs (A_*ij*_ = 1) and *r*_0_ for the unconnected pairs of regions (A_*ij*_ = 0), respectively (Fig 1B) (see Material and methods). The maximal recovery occurs at α = 0.006 (*r*_1_ = 0.67, *r*_0_ = 0.91), which is better than the recovery only for efficiency optimization at α = 0 (*r*_1_ = 0.45, *r*_0_ = 0.84) or only for wiring cost optimization at α = 1.0 (*r*_1_ = 0.61, *r*_0_ = 0.89). Both recovery rates are clearly higher than that (*r*_1_ = 0.52, *r*_0_ = 0.80) for the synthetic network produced by the original generative model [14] with the optimized parameters but without fixing degrees, and are also higher than that (*r*_1_ = 0.56, *r*_0_ = 0.82) for extended generative model with fixed degrees. Since the extended generative model and the cost-efficiency model both have the same degree sequence as the real network, further comparison will be performed between these two models. Optimizing the wiring cost by itself at α = 1 can recover most of the short-distance connections (*r*_1_≈ 1 for small distance, Fig 1C) while suppressing the long-distance connections (*r*_0_≈ 1 for large distance, Fig 1D). However, by combination with the processing efficiency constraint at α = 0.006 (see the Discussion for why such a small value is needed), the model network can clearly better recover the long-distance links (Fig 1C, larger *r*_1_ at long distances, e.g., x > 30 mm, especially as in the two longest bins in dashed circle) and also avoid false-positive links of pairs in the spatial neighborhood (Fig 1D, larger *r*_0_ at shorter distance, e.g., x < 10 mm). Changes of the recovery rates *r*_1_ for long distance connections (> 30 mm) and *r*_0_ for short-distance pairs (<30 mm) with respect to α in S2 Fig showed that the improvement of the recovery of long distance links is quite significant, although the absolute increase of the recovery rate *r*_1_ from 0.61 to 0.67 appears not as a big change. In comparison, the cost-efficiency trade-off model has a clearly higher recovery rate (larger *r*_1_) and fewer false-positive links (larger *r*_0_) than the generative model (Fig 1C and 1D). The cost-efficiency trade-off model better recovers the middle-range connections and performs especially well in avoiding the false-positive links at short distance, which clearly reflects the competition of wiring cost and processing efficiency, respectively, that is associated with short and long-distance connections.

These results show that efficient processing requires long-distance connections, but the real network possesses even more long-distance connections than the best trade-off model provides, suggesting that further functional factors are at work in the real network.

### Good recovery of multiple statistical features of the connectome by the cost-efficiency model as well as by the generative model

**Fig 2:**
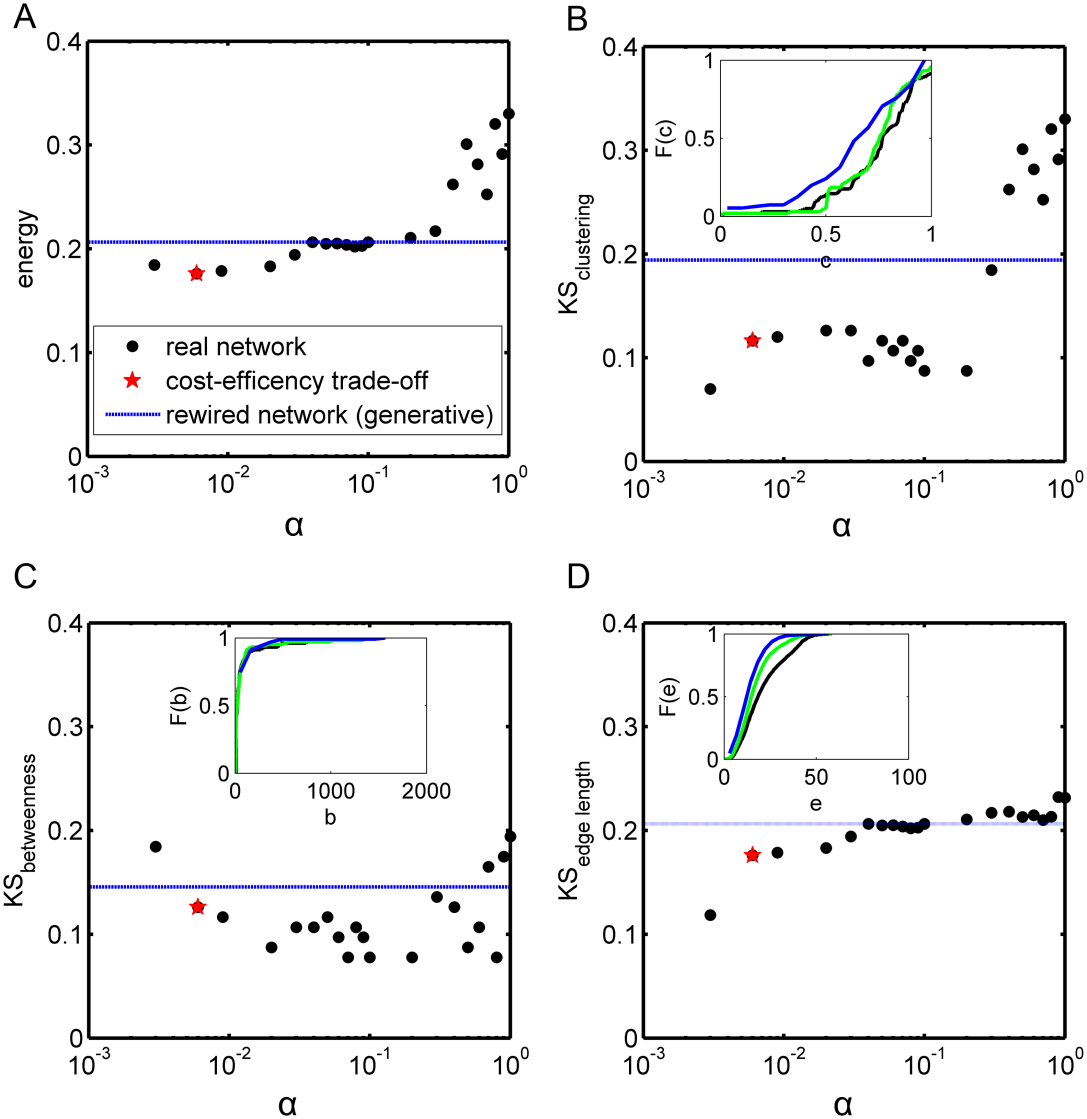
The recovery of different statistical features of the real networks by the cost-efficiency trade-off model and the optimal generative model. (A-D) show the behavior of energy (A), Kolmogorov-Smirnov statistics *KS*_*clustering*_ (B), *KS*_*betweenness*_ (C), and *KS*_*edge length*_ (D) as a function of α (bullets) by the cost-efficiency trade-off model. The red stars represent the corresponding results at α = 0.006. Blue dash lines show the corresponding results for the optimal generative model with fixed degrees. Inset in (B), (C) and (D): Cumulative distribution of clustering coefficient, betweenness centrality, and edge length for the real cortical network (black line), for the cost-efficiency trade-off model at α = 0.006 (green line), and for the generative model with fixed degrees (red line).

In previous work using a generative model with link-generating rules combining spatial distance and topological similarity (i.e., matching index), several different topological features were well recovered for the brain networks of different species [14, 24, 25]. Here we also applied statistical measures to quantify the performance of this generative model on the *macaque* connectome with optimized energy (see Methods), and compared the outcome with reconstructed networks produced by the cost-efficiency trade-off model across different weight parameters α (Fig 2).

For the *macaque* cortical network, the synthetic network of the original generative model [14] with optimal parameter values was obtained by combining the spatial distance and the matching index, achieving the minimal energy E = 0.14, which was quite similar to the previous result for the human connectome [14]. As for the extended model with fixed degrees, the minimal energy E = 0.2. This is plausible since under the additional constraint of fixed degrees the generative rules may have reduced freedom to recover the other statistical features. As for the cost-efficiency trade-off model, the energy E clearly decreased with α, reaching a minimum at α = 0.006 and the E value was very close to that of the optimal generative model (Fig 2A). The result of the similarity of each statistical measure of clustering, betweenness centrality and edge length shown in Fig 2B–2D provides further support that the best trade-off model performs as least as well as, generally slightly better than the optimal generative model (fixed degrees). The cumulative distributions of the three measures were similar for the real cortical network, the optimal generative model and the best cost-efficiency trade-off model (insets of Fig 2B, 2C and 2D).

Taken together, the results showed that a proper trade-off between cost and efficiency not only better recovers the individual links, but also simultaneously reproduces multiple statistical features of the real network as done by the generative model which explicitly targets such measures as its objective function.

### Recovery of connectivity related to functional organization and spatial layout

**Fig 3:**
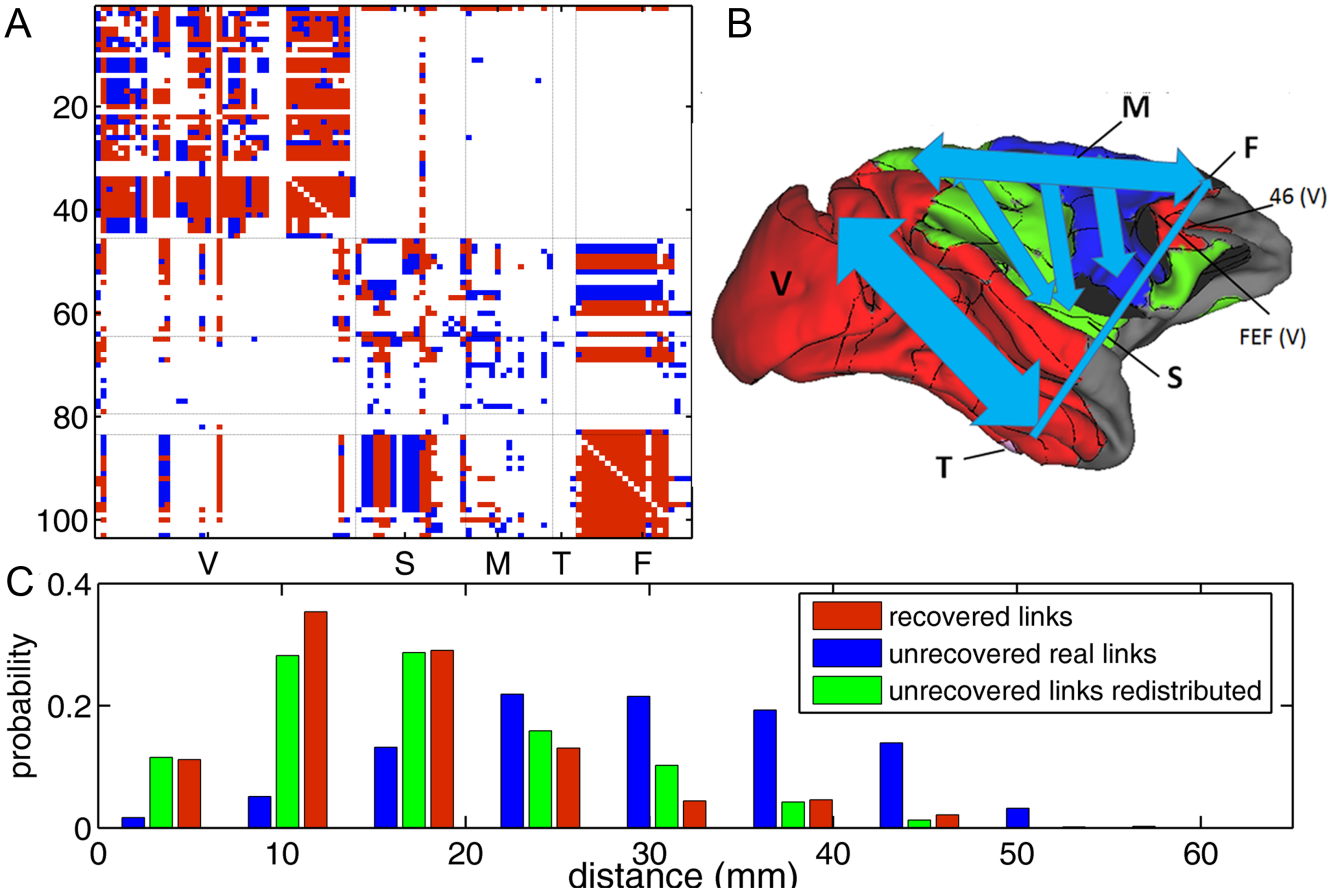
Recovered and unrecovered links by the trade-off model. (A) The adjacency matrix *A* of the real network. The red and blue color respectively represent links that are recovered and not recovered by the reconstructed network at α = 0.006. The cortical areas are grouped by functional systems. (B) Schematics of the distribution of unrecovered links (blue) in the real network. The width of the arrows (roughly) indicates the number of connections. The functional systems are denoted by color: visual (red), somatosensory (green), motor (blue), temporal (pink) and frontal (black). The cortical surface is constructed by the CARET software. (C): Probability distribution of the recovered (red), unrecovered (blue) links in the real networks vs. the spatial distance. The green bars show the distribution after the blue links are rewired in the reconstructed networks.

Recovery of connectivity by the trade-off model is not uniform and appears related to the functional network organization and spatial layout (Fig 3). Connectivity within the group of visual regions and within the whole frontal cortex is almost fully recovered (Fig 3A, red). There is also a significant portion of inter-modal links that can be recovered (mainly between frontal and other sub-systems). On average, 72.5% of links within functional systems can be recovered, but the links between functional systems have a lower recovery rate of 56.3%. The recovered links are typically short-distance (Fig 3C, red bars).

Overall, there are 33% links in the real network that cannot be reliably recovered with the best cost-efficiency trade-off model (blue links in Fig 3A). The unrecovered links are mainly found within the visual system (38.6%) and between the somatosensory and frontal systems (25.6%); see Fig 3B for schematics of the distribution of unrecovered (blue) links in the real network. The unrecovered links are typically long-distance (Fig 3C, blue bars). In the reconstructed network, these unrecovered links are redistributed (see S3 Fig for the adjacency matrix $\bar{A}$ of the reconstructed network and the schematics of redistribution of unrecovered links on the cortex), typically becoming short-distance links (Fig 3C, green bars). Visual inspection of Fig 3A suggests that these unrecovered links (blue) are not uniformly distributed in the connectivity matrix, but are mainly concentrated on a few regions in the visual and somatosensory systems.

To investigate the region-dependent recovery, we obtained the node recovery rate

**Fig 4:**
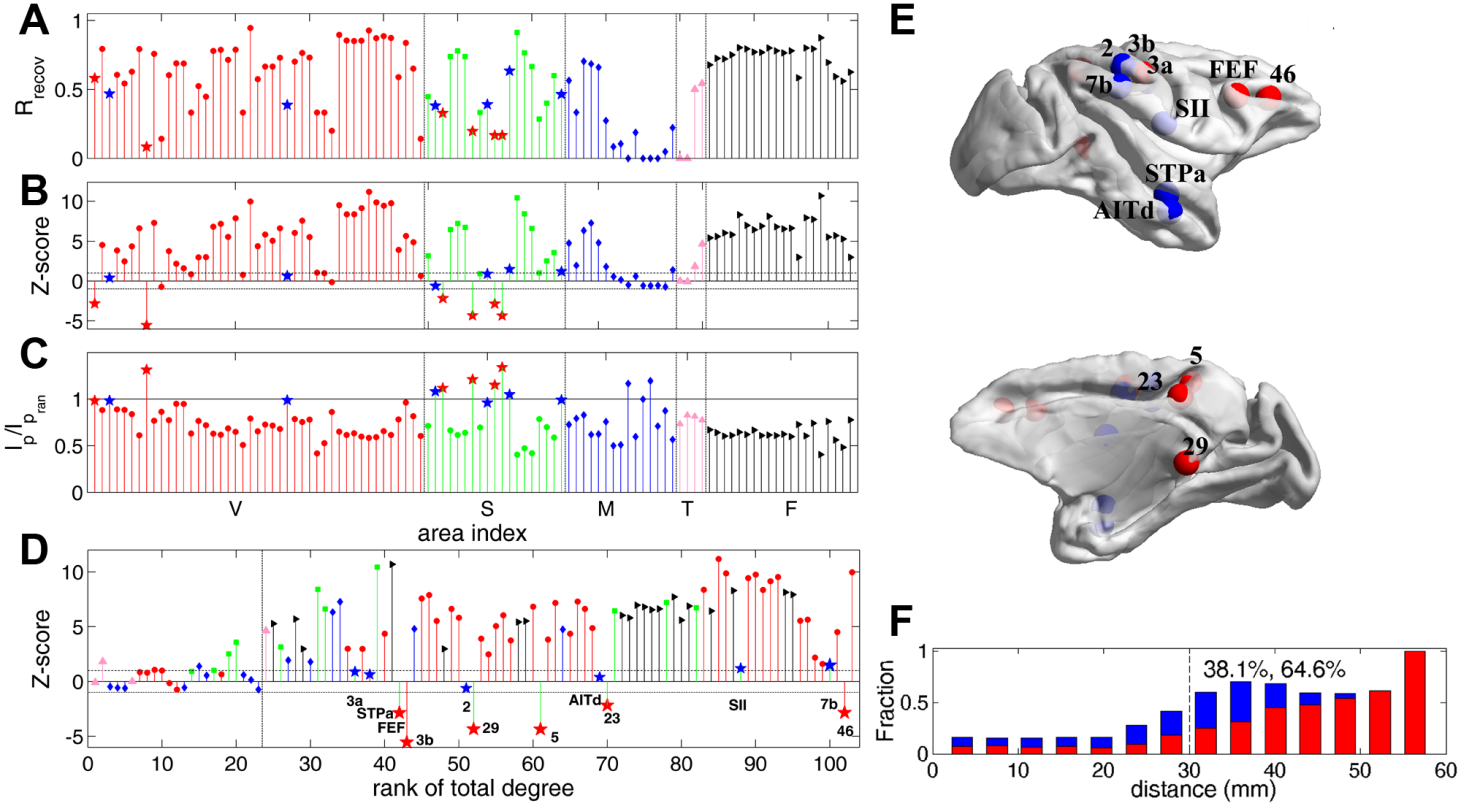
The structural properties of LDCs. (A) Recovery rate *R*_recov_ for each area. (B) The Z-score of the recovery rate Z_*R*_ (*i*) of the reconstructed network (α = 0.006) when compared to the random benchmark networks. (C) The total wiring length *l*_*p*_ (*i*) of all the links of an area in the real network with respect to the corresponding total wiring length *l*_*pran*_ (*i*) (average) in random networks. (D) The Z-score of the areas sorted by the rank of total degree. The vertical dashed lines in (A-D) indicate the separation of the functional systems (visual (V): red; somatosensory (S): green; motor (M): blue; temporal (T): gray and frontal (F): black). The horizontal dashed lines in (B) and (D) indicate the range of Z-score in [− 1.65, 1.65]. The vertical dashed line in (D) separates the areas to the left with small degree and insignificant recovery. (E) Spatial location of LDCs on the *macaque* cortex using BrainNet Viewer [81]. (F) The proportion of the links in each distance bin occupied by the six core areas (red bars) and the other six special areas (blue bars). The dashed line is a rough boundary between short-distance (< 30 mm) and long-distance (> 30 mm) links. Overall, the six core areas involve 38.1% and the 12 special areas involve 64.6% of long-distance links in the real network.

*R*_recon_(*i*) of the total links (input and output) of each region *i* by the reconstructed network (Fig 4A). We further quantified the significance of *R*_recon_(*i*) with respect to that of random networks by the Z-score (Fig 4B). Z_*R*_ (*i*) > 1.65 (corresponding to p<0.05) means that the reconstructed network shows a significant recovery of real links of a region relative to coincident recovery in random networks. Regions with low recovery rate in Fig 4A in general have an insignificant Z-score in [−1.65, 1.65]. From Figs 3A, 4A and 4B, we observed the following features. (*1*) All frontal regions have high and significant link recovery rate, and only links with some somatosensory regions cannot be recovered (Fig 4A); (*2*) Most of the visual regions have Z_*R*_ (*i*) > 1.65. The majority of the unrecovered links of visual regions are within the visual system, except for a few links with frontal or somatosensory regions (Fig 4A); (*3*) Several somatosensory, temporal and motor regions have poor and insignificant recovery rate (Z-score in [−1.65, 1.65]). The insignificant recovery rate of regions is mostly due to a small total degree: 18 regions with Z-score in [−1.65, 1.65] are within the bottom 22 regions ranked by total degree (left to the vertical dashed line in Fig 4D, degree < 20).

However, the overall recovery rate for the regional connectivity profiles is quite low for many regions in the optimal generative model (S4A Fig). Apart from the insignificant recovery rate of regions with degree <20 as in the cost-efficiency trade-off model, there are 33 regions with Z_*R*_(*i*) <1.65 (p>0.05) (S4B Fig), whose connectivity cannot be recovered by the generative model better than coincidence in random benchmarks (all with the same fixed degrees). This number is almost three times as the number 12 of the cost-efficiency trade-off model (Fig 3). Specifically, the recovery rate for the hub regions is high with a large Z-score in the cost-efficiency trade-off model (Fig 4D), but is quite low for the generative model (S4C Fig). Since the cost-efficiency trade-off model not only better recovered the individual links (Fig 1), but also the regional connectivity profiles (Fig 4), the analysis in the following sections focuses on the cost-efficiency trade-off model.

### Special regions with a connectivity profile unexplained by cost-efficiency trade-off

Interestingly, there are six regions with Z_*R*_(*i*) < − 1.65 in Fig 4B (denoted by red stars), indicating that the connectivity of these regions in the real network is quite special: the best cost-efficiency trade-off model performs significantly worse than random coincidence for recovering the actual links of these regions. We systematically identified such regions with Z_*R*_(*i*) < − 1.65 in the real network when compared to model networks at different α. It was found that the number of such regions is minimal at α = 0.006 where the overall recovery rates *r*_1_ and *r*_0_ are maximal (Fig 1A), while it is maximal at α = 1 (S5A Fig). The total number of regions with insignificant recovery rate (Z_*R*_(*i*) < 1.65) also shows a minimum at α = 0.006 (S5B Fig).

The six core regions in the real network whose connectivity profile is unexplained by cost-efficiency trade-off, identified by comparison with the best trade-off model at α = 0.006, are visual (46, FEF) and somatosensory (23, 29, 3b, 5) regions. They are also among the regions with Z_*R*_(*i*) < − 1.65 at different α. Except for region 46, five regions have intermediate degree ranks (Fig 4D, red stars, 41 < degree < 70). In total, there are 381 links from these six core regions, but they contribute 266 (31.4%) of unrecovered connections. In Fig 4D, we also marked out six more regions (2, AITD, STPa, 3a, SII and 7b), having intermediate or large degrees, but with a recovery rate not significantly lower than for random benchmarks (− 1.65 < Z_*R*_(*i*) < 1.65), by blue stars. Together with the former core six regions, these 12 regions (see Fig 4E for the positions) with intermediate or large degrees but poor recovery contribute about 60% of the unrecovered links.

**Fig 5:**
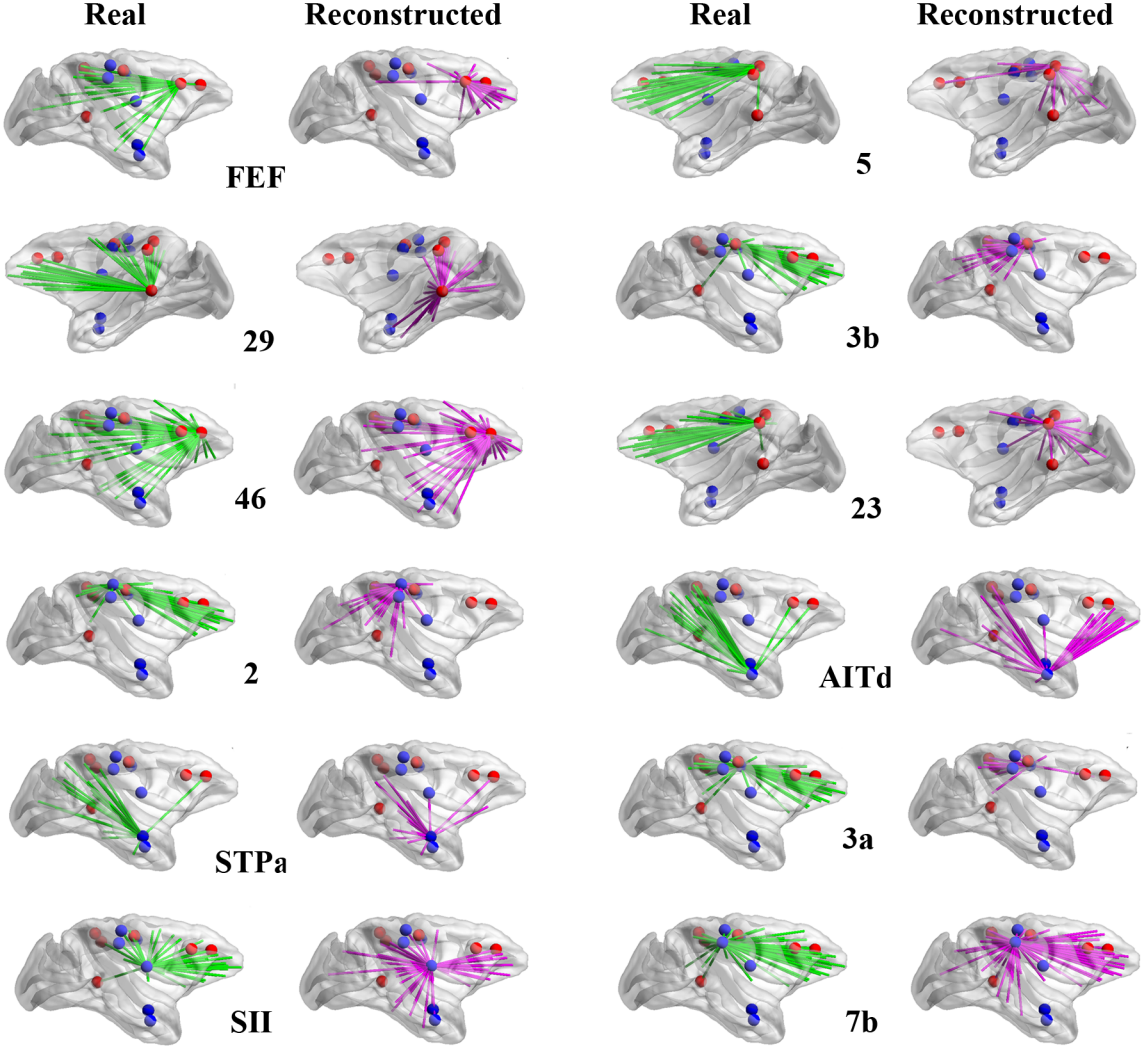
Comparison of the connections of 12 LDCs areas with Z_*R*_(*i*) < 1.65. The green and pink lines represent the connections of the area in the real network and the reconstructed network, respectively. The values are mapped on the cortical surface using BrainNet Viewer [81]. The name of the area is indicated in the plot. The six core LDC areas are marked in red dots, while the remaining six LDCs are marked in blue dots.

The connectivity of the six core regions and the other six regions in the real network and in the best cost-efficiency trade-off model at α = 0.006 is shown in Fig 5. In the real network, these regions tend to connect with remote targets, but in the reconstructed network they mainly connect to spatially neighboring regions. Therefore, for all the 12 regions, the wiring length *l*_*p*_ in the real network is large, very close to, or even larger than *l*_*pran*_ in the random networks (Fig 4C, *l*_*p*_/*l*_*pran*_ ≳ 1 for the stars). Strikingly, the six core regions involve almost all of the most distant connections in the real network (Fig 4F, red bars) and the other six regions occupy a significant portion of intermediate-to-distant links (Fig 4F, blue bars). Overall, the six core regions and the 12 regions contribute 38.1% and 64.6% of the long-distance links (x > 30 mm), respectively. The above analysis clearly shows that the real network possesses more long-distance links and that they are distributed highly non-uniformly, concentrated on few regions, forming long-distance connectors (LDCs).

**Fig 6:**
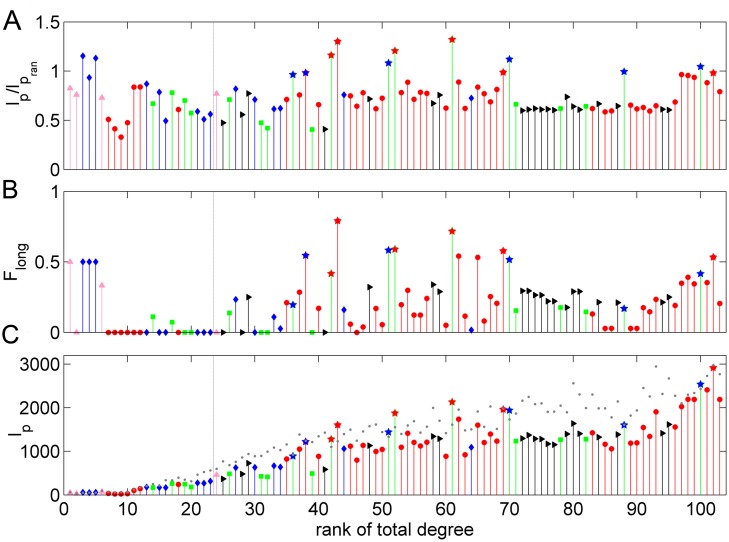
Regional structural properties by the rank of total degree. (A) The total wiring length *l*_*p*_ (*i*) of all the links of an area in the real network with respect to the corresponding total wiring length *lpran*(*i*) (average) in random networks by the rank of total degree. (B) The fraction of long-distance connections to all the links for each area sorted by the rank of total degree. (C) The total wiring length *l*_*p*_(*i*) of all the links of an area in the real network. The corresponding total wiring length *l*_*pran*_ (*i*) (average) in random networks by the rank of total degree from small to large, marked by the grey dots. The colors indicated the functional systems (visual (V): red; somatosensory (S): green; motor (M): blue; temporal (T): gray and frontal (F): black). Note that LDCs (stars) with intermediate degrees have a total wiring length comparable to areas with the largest degrees (hubs).

Previous studies have focused on hubs with a large number of connections [12, 32, 35, 36]. Interestingly, however, the ratio of the wiring length *l*_*p*_ of the hubs in the real network to *l*_*pran*_ in the random network, which is just around 1, is not as high as for the LDCs (Figs 4C and 6A). Especially for the input connections, most hubs have relatively low *l*_*p*_/*l*_*pran*_ (~65%). Although the hub regions have a large number of connections, the long-distance links represent only a small fraction of connections for the hubs (< 0.4, Fig 6B). Thus, the total wiring distance for the hub regions is more or less the same as for the LDCs, some of which is even less than LDCs (Fig 6C).

### LDCs underlie hierarchical modules and a dense network group for functional segregation and integration

What could be the functional impact of the LDC regions which strongly violate the wiring cost economy of the network? In neural systems it is desirable to achieve a balance of functional segregation and integration [37, 38]. Network substrates providing such a balance may be densely connected modules which are not too strongly affected by other systems but are still properly interlinked by sparser connections among the modules [39, 40]. More generally, a hierarchy of modules can provide segregation and integration across scales of organization [41]. Here, in order to explore the functional influence of the LDCs, we compared the functional segregation and integration of three types of networks. They were the real biological network, the reconstructed network (at α = 0.006) with only about 30% of total links different from the real network, and the R-network (see Methods) in which the concentration of long-distance links on LDCs was destroyed, but the wiring cost was the same as in the real network.

#### A. The rich hierarchical network structure does not exist when the concentration of long-distance connections to LDCs is destroyed

**Fig 7:**
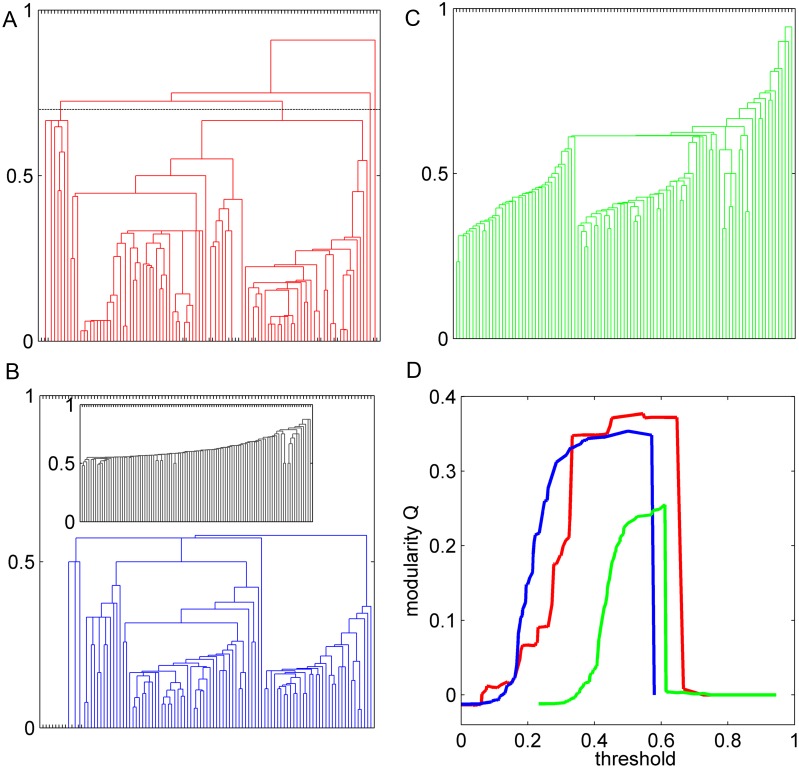
Comparison of hierarchical modular organization of the real and surrogate networks. Hierarchy trees for the real network (A), reconstructed network (B) and R-network (C) where the unrecovered links of LDCs in the original network are rewired while keeping the wiring cost. Inset of B: random network rewired from the real network while keeping the degrees. (D) Modularity Q as a function of the threshold values for the trees in A, B and C (corresponding colors).

We obtained hierarchical trees (dendrograms, see Methods) for the three networks (Fig 7A–7C). Applying a threshold value to the hierarchy tree (e.g., indicated by the horizontal dashed line in Fig 7A), we obtained module partitions, and computed the modularity Q [41] for the partition at different threshold values (Fig 7D). Interestingly, the real network allows for a very complex hierarchical modular structure across many scales (levels of resolution), corresponding to several flat steps and jumps of Q in a broad range of thresholds (Fig 7D, red line). The best trade-off model (without many long-distance connections) still showed some large modules at large thresholds (Fig 7B), but submodules were destroyed (the trees inside some large modules are very similar to the random network in the inset), and Q changed continuously without clear steps and jumps (Fig 7D, blue line). Basically, the reconstructed network has a prominent modular organization only at one level (one relatively flat regime of Q around threshold ∼ 0.5). In the R-network, which preserves most links and total cost as in real network but randomizes unrecovered connections of LDCs (mostly long-range connections), there appear to be two large modules at a narrow range of thresholds (∼0.6, Fig 7C and 7D, green line), but the trees within the modules are very similar to random networks. The maximal modularity Q in the real network is larger than that for the other two networks. Thus, the real network allows for a complex hierarchical modular structure across a large range of scales when compared to the reconstructed and R-networks.

#### B. LDCs allow proper functional segregation

**Fig 8:**
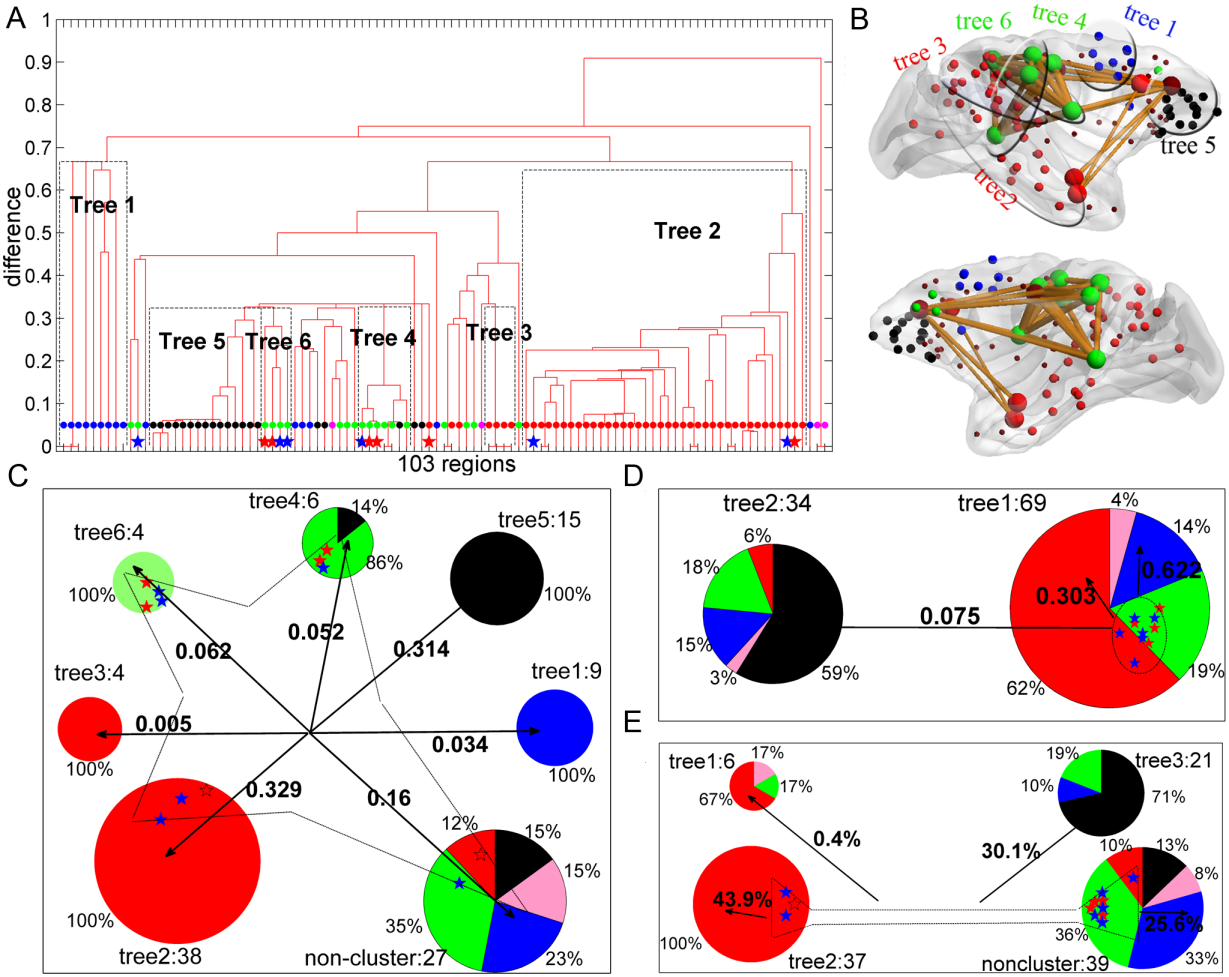
The LDCs affect the functional segregation and integrations. (A) Hierarchical tree of the real *macaque* cortical network. The color of dots represents the corresponding function (visual: red, somatosensory: green, motor: blue, temporal: pink and frontal: black). The red stars indicate the 6 core special areas of LDCs, and the blue stars for the rest 6 LDCs. (B) Spatial distribution of different sub-trees in the real network mapped on the cortical surface using BrainNet Viewer [81]. The LDCs in the sub-trees are shown as big balls with connections among each other. (C, D, E) Hierarchical modular organization of the cortical connectome, the reconstructed network at α = 0.006, and a randomized network (‘R-network’), respectively. Every pie in (C), (D) or (E) represents a hierarchical sub-tree (connectivity module) dominated by areas of a certain functional modality (matching ratio > 50%) in the corresponding network. The number of areas is listed next to the tree. The areas not involved in any hierarchical tree are combined into the “non-cluster” pie. The colors in the pie represent different functional systems (as in Fig 3B and 3A), and the corresponding ratios of these systems in a given hierarchical sub-tree are listed. The original LDCs belonging to different sub-tree or non-cluster groups are shown by stars. These areas are grouped together by light solid lines with a diamond shape for the real network (C), by the shape of the circle in the reconstructed network (D), or by the shape of a hammer in the R-network (E). The arrows from the group of the special areas indicate the distribution of the links of this group to other sub-trees, with the ratio listed by bold fonts near the corresponding arrows.

We performed a detailed analysis of the matching between the connectivity modules represented by the subtrees and the actual functional cortical divisions (see Methods). We first considered the hierarchical tree of the real network in Fig 7A. At least five functional systems of the *macaque* cortex can be recognized [2, 3], which are visual, somatosensory, motor, temporal and frontal divisions (as shown in Fig 3). In the real network, the regions within each subtree are spatially clustered (Fig 8B), thus, they have a smaller spatial distance and denser connectivity than those between sub-trees (S6 Fig). If one sub-tree has a strong overlap with specific functional subdivisions, without being mixed with many other functional systems, then this dense module is able to strongly support the specialized functional modality of the local regions. The matching between network modules and functional subdivisions is graphically presented in Fig 8A–8C for the real network. Fig 8A shows that the connectivity in the real network provides network substrates for functional segregation. Except for the temporal cortex with only 4 regions [42], each functional system (visual, somatosensory, motor and frontal) has at least one supporting modules (subtree), and the visual and somatosensory systems are maintained by two subtrees (the fraction of functional regions taken by the trees is listed in S2 Table). Five sub-trees have a 100% match with a particular function, and only one sub-tree (Tree 4 in Fig 8A–8C) has one mismatched region. Together, the six trees having a high match with one of the functional subsystems represent about three quarters (72.7%) of the total cortical regions. The remaining regions are not clustered into a tree with a dominant function, and were accordingly assigned to the “non-cluster” pie in Fig 8C.

The same procedure was applied to the reconstructed network (at α = 0.006), in which one third of the intermediate and long-distance connections are rewired into short-distance connections (Fig 3), and R-networks, in which the links of LDCs are randomized while keeping the same distribution of physical distance, so that the concentration of long-distance links on the original regions of LDCs is destroyed. In the reconstructed network, there are only two trees showing dominance by the regions in the original visual and frontal systems, but each connectivity module is mixed with many regions from other functional systems (Fig 8D). This is due to the fact that the somatosensory and motor regions are either densely connected with the visual or frontal systems after reconstruction. In the rewired R-network, 3 trees are obtained with dominance by the visual and frontal systems, but two trees are strongly mixed with many other functional regions (Fig 8E). In both reconstructed and R-networks, the approximate separation of the original visual and frontal systems into different modules is due to the spatial separation of these two divisions and based on short-range connections within each division (2/3 of the links, Fig 3A, red, are recovered in the reconstructed network and are common in the three types of networks analyzed here). Thus, the segregation into 5 functional subdivision cannot be properly realized by the network connectivity in the reconstructed and R-networks. This is surprising, because R-networks and the real cortical network share at least 80% of common, short-range links.

#### C. LDCs have high functional diversity coefficient and form a dense group for functional integration

**Fig 9:**
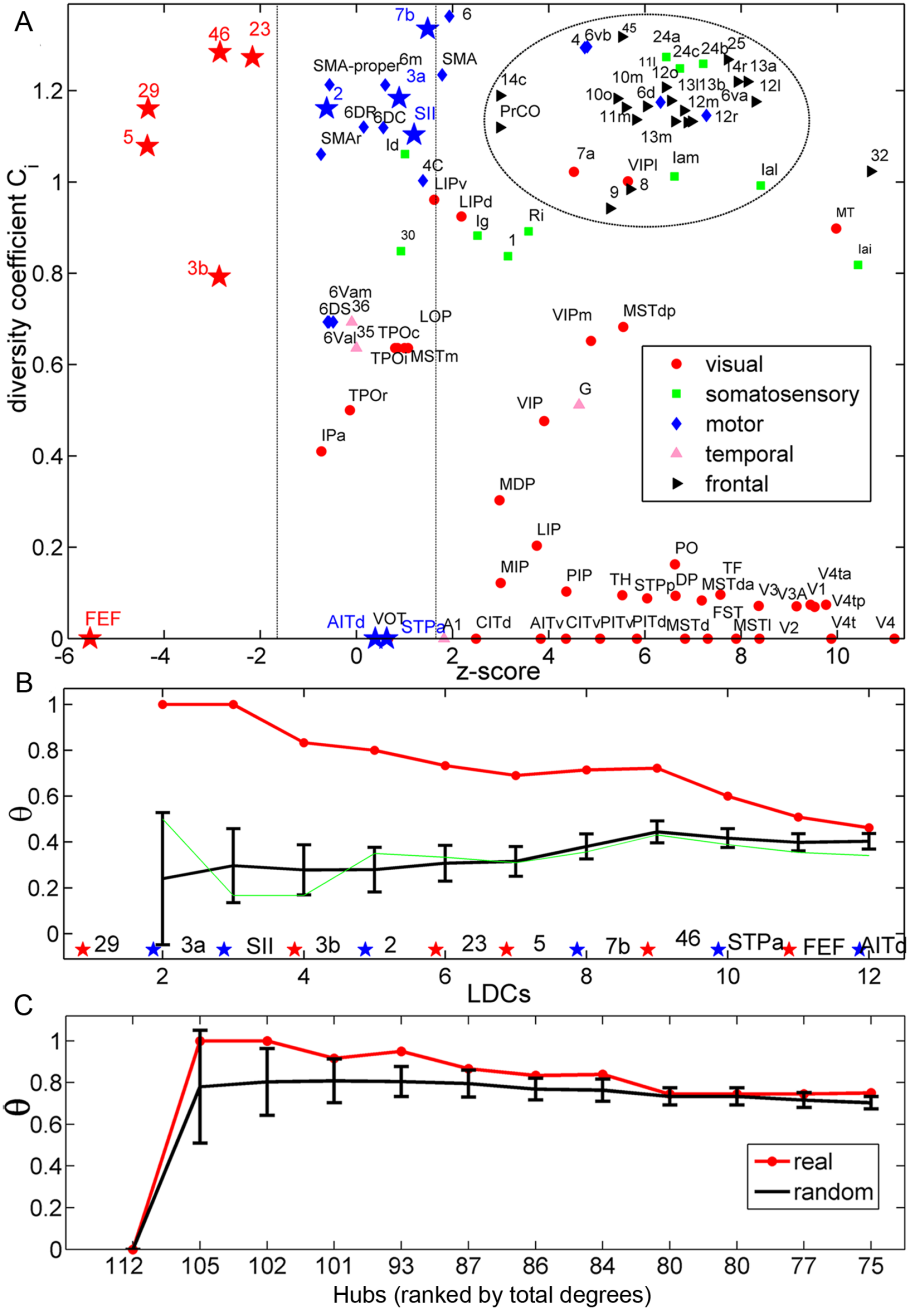
Functional integration impacted by LDCs. (A) Diversity coefficient across different functions vs. the recovery Z-score of each area by the best trade-off model (α = 0.006). The vertical dashed lines indicate the range of the Z-score in [− 1.65, 1.65]. (B) Dense group index θ for groups of nodes formed by different sizes of the LDCs (red and blues stars shown above the x-axis, ranked by the density for LDC groups at different sizes) for the connectivity in the real network (red) and in the R-network (green). The black line and error-bars represent the average value and standard deviations of the density in an ensemble of 100 corresponding random networks. In the subgraphs, the six core LDC areas are marked as red stars and the remaining six LDC areas are marked as blue stars. (C) Rich-club index θ for hub nodes ranked by the total degree (from high to low) for the connections in the real network (red). The black line and error-bars represent the average value and standard deviations for an ensemble of 100 random networks rewired from the real network while keeping the degrees.

We examined how the 12 LDC regions are distributed among the different hierarchical sub-trees. In the real network, the 12 LDC regions are placed in different sub-trees (Fig 8A and 8C), seven in two somatosensory trees, three in one of the visual sub-trees and the other two in the non-cluster pie. Notably, most of the LDC regions either appear near the boundary of a hierarchical sub-tree, or between different sub-trees (Fig 8A), implying that they have a large proportion of connections with other trees and play a role in the communication among different functional modalities. The role of different cortical regions in inter-modal interactions was quantified by the distribution of the links of a region among different functional regions in the real network, using the functional diversity coefficient *C*_*i*_ of a region (see Methods).*C*_*i*_ of the real network is shown with respect to the Z-score of the recovery of the real links by the best trade-off model at α = 0.006 (Fig 9A). The 12 LDC regions typically show high functional diversity coefficient (Fig 9A, red and blue stars), except for frontal and temporal regions FEF, AITd and STPa. These three visual regions have *C*_*i*_ = 0 since they only form connections within the visual system. However, these regions mainly have long-distance connections to spatially distant visual regions, and such distant connections cannot be well recovered by the trade-off model. It is important to stress that although many LDC regions (3a, STPa, FEF, 3b, 2, 29, 5, AITd and 23) just possess average degrees (Fig 4D), the links of these regions are distributed uniformly across different functional modalities, and their diversity coefficient is comparable to the real hub regions 7b and 46. Apart from the 12 LDC regions, frontal regions (mostly staying in the dashed circle at the top right part of Fig 9A) have high *C*_*i*_, since they connect within frontal, and with motor, somatosensory and temporal systems (Fig 3A), and these systems are spatially close to the frontal system (Fig 3B). Such economic links can also be well recovered (large Z-score). As for some motor regions, the high *C*_*i*_ could be attributed to the fluctuation from the small degrees (Fig 4D).

From these observations one can conclude that the LDC regions act as links among spatially segregated and specialized functional regions. LDCs also form densely connected groups (red line in Fig 9B, see Method for the dense group measure θ), a structural substrate for integrating information collected by these regions from segregated functional sub-systems. The dense group connections among LDCs are shown in Figs 8B and 9B. We examined how the whole dense group of 12 LDCs distributes links to different functional subtrees (Fig 8C). Most (84%) of the external connections of the dense group are distributed across the six functional trees (e.g., 33.4% of connections with two subtrees of visual regions, 31.4% with the subtrees of frontal, 11.4% with two subtrees of somatosensory, and 3.4% with motor). Thus, the dense group of LDC regions overlaps on the subtrees, to integrate information of the functional modules. Previous study revealed that the visual system possesses a hierarchical structure with multiple (10) levels [2], starting from cortical areas V1 and V2 staying at the bottom levels as input regions. As a direct evidence of the role of functional integration of LDCs, five of them (FEF, STPa, AITd, 7b and 46), which are involved in visual processing, are regions all staying at high levels (at the top three levels which are composed of a total of 13 regions) of visual information processing flow [2]. This finding of a dense group of LDCs with intermediate node degree is quite different from previous analyses of rich-clubs formed by degree-rich nodes [32]. The conventional rich-club only includes the seven biggest hubs (MT/V5, 46, 7a, 7b, LIPv, LIPd, VIPl) with the connection density among them just slightly beyond one standard deviation of the random benchmarks (Fig 9C), not as significant as the dense group among LDCs (Fig 9B). When the concentration of the long-distance links on LDCs is destroyed in the R-network, these regions no longer form a dense group (Fig 9B, green line). In summary, the topological features of LDCs suggest that they are essential for both functional segregation and integration in the cortical network.

### Reliability of the findings of LDCs

The primate cortical network analyzed in the present study was based on a previous dataset collated from the anatomical literature [18, 43], and there could be concern that the connections of some cortical regions may be incompletely characterized. Recently, a more systematic tract-tracing compilation of cortical connectivity of the non-human primate brain, including the relative weights of fiber projections was published, but only a partial square matrix dataset for 29 out of 91 targeted regions was presented [44, 45]. While this relatively small dataset is not quite suitable for the trade-off modeling approach, it still allowed us to assess the reliability of our main findings (see Material and methods).

**Fig 10:**
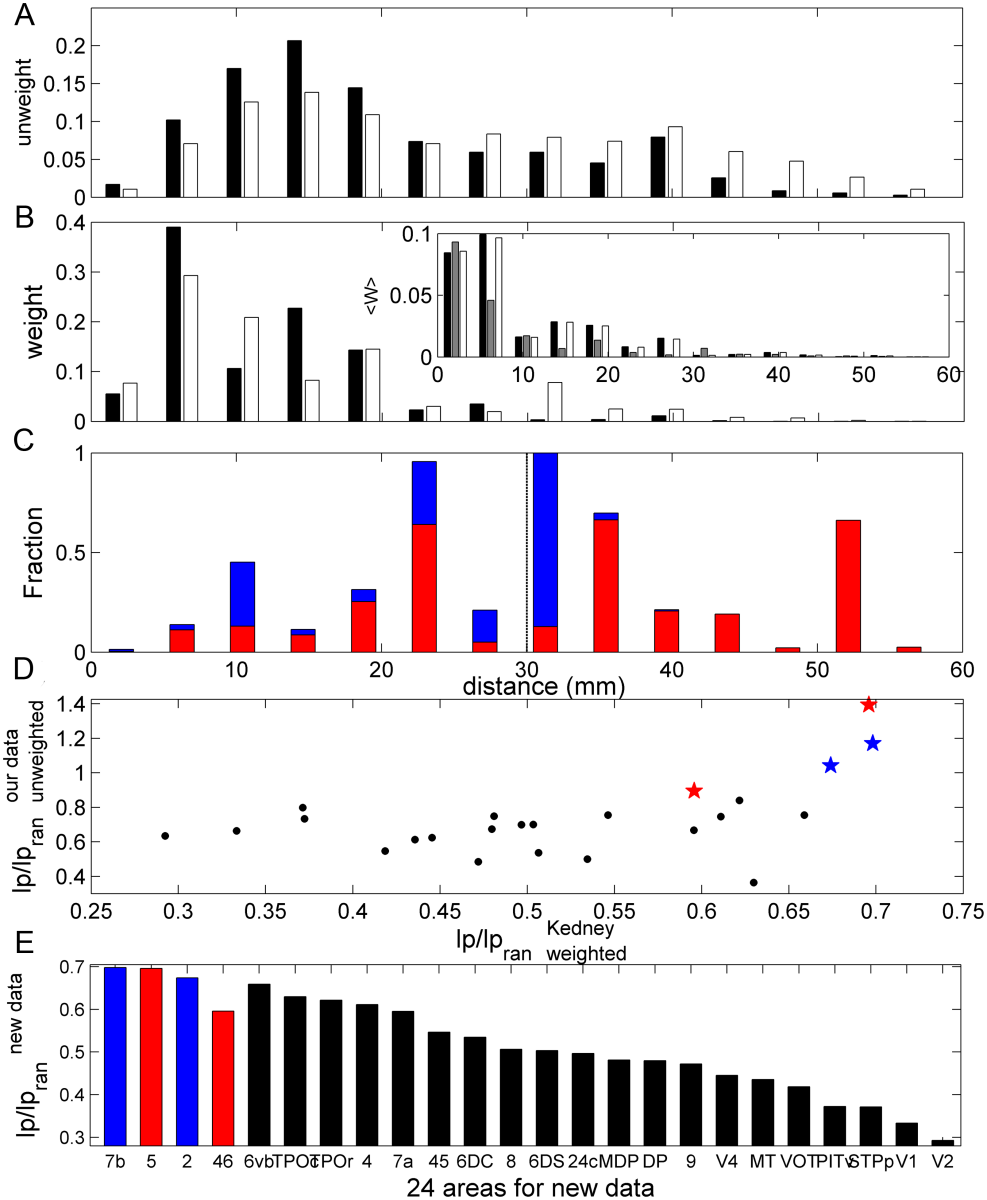
Comparison between the data of [45] and the present dataset. (A) Distribution of (binary) links with respect to distance between cortical areas for all the links in data of [45] (white bars) and the links overlapping with our data (black bars). (B) As in A, but for the distribution of the projection weights. The inset shows the average projection weight vs. distance. Here the gray bars are for the new links in [45] non-overlapping with our data. (C) The portion of the total projection weights within each distance bin in the data of [45] occupied by the two core LDC areas (5 and 46, red bars) and the other non-core LDC areas (2 and 7b, blue bars). (D) The total projection cost of each of the 24 targeted area in [45], with respect to that in the corresponding randomized networks, weighted l_*p*_/*l*_*pran*_, is compared to the corresponding l_*p*_/*l*_*pran*_ from the present data (binary global network, similar to Fig 4C, but only concerning the afferent direction here). The red stars show the 2 areas of the data of [45] (5 and 46) appearing in the 6 core LDC areas in our data. The blue stars show the 2 areas of [45] (2 and 7b) appearing in the non-core LDC areas. (E) Each bar corresponds to the weighted ratio l_*p*_/*l*_*pran*_ for each of the 24 targeted areas in new dataset [45]. The first 4 bars refer to the 4 LDC areas in our data, which is ordered by the value of l_*p*_/*l*_*pran*_. The following 20 areas are also ordered by the l_*p*_/*l*_*pran*_ values.

First, the primary dataset and the Markov et al. dataset [45] are consistent in different aspects when considering the overlapping subsets of nodes. *(i)* Most links within functional systems largely overlap in two datasets. However, the new dataset revealed more intermediate and long distance connections (Fig 10A), which mostly consist of links among different sub-functions, e.g., between visual and frontal systems; *(ii)* the average weight for the links of a given physical distance decays with the distance for the links found in both datasets and the links overlapping with the present dataset appear much stronger in weight than those newly found links revealed in [46] (Fig 10B). This comparison confirmed that our dataset collated from the literature is reliable when compared to the more systematic new dataset, since it captured the significant and strong projections. The weaker links revealed in the new dataset would not make much contribution to the overall wiring cost in terms of neuron-to-neuron projection.

Next, we examined whether the observed concentration of the long-distance links on a few LDC regions from the global network of our dataset, i.e., the formation of LDCs, is also apparent in the new dataset. In particular, 4 of the 12 LDCs were examined in [45], including 2 core LDC regions (5 and 46) and non-core LDC regions (2 and 7b). Similar to Fig 4F, we checked how much fraction of the projection weights in the distance bin was concentrated on these 4 regions in the new dataset (Fig 10C). It is very interesting to see that, although the new dataset contains only partial connections (i.e., only the input projections), the distribution pattern in Fig 10C is very similar to that of the global network in our data in Fig 4F: the two core LDC regions 5 and 46 (1/12 of the 24 target regions), occupy 21.5% of the weight of long-distance projections (> 30mm) in [45]. The other two regions, 2 and 7b, occupy a significant portion of intermediate-distance projections (20 ∼ 35 mm). In all, the 4 regions (1/6 of 24 regions) contribute 54.8% of the weight of long-distance projections (> 30 mm).

It is shown in Fig 4C in our global dataset that for the LDC regions, the total connection cost *l*_*p*_ of the region in the real network with respect to that in the corresponding randomized networks, l_*p*_/*l*_*pran*_, is close to, or larger than, 1. In the Markov et al. data [45], we first calculated the wiring cost, then calculated a similar weighted ratio l_*p*_/*l*_*pran*_ after incorporating the projection weights (see Methods). In this case, the weight value of links to the same targeted region is comparable and reflects the projection density from different sources. The wiring cost in the new dataset was calculated by the weighted distance. Since weight information reflects the number of fiber projections, the weighted distance more accurately describes the corresponding wiring cost. However, the weighted network has incomplete cortical coverage (only about 1/3 of the cortex); thus, we can only check the consistency by comparing the weighted ratio for the 24 targeted regions to the ratio in our dataset (Fig 4C) which was obtained from binary global networks, as shown in Fig 10D. Interestingly, the ratio l_*p*_/*l*_*pran*_ of most regions in our data is proportional to that in [45]. Except for region 46, the remaining 3 LDC regions 5, 2 and 7b appearing in the 24 targeted regions in [45] all have the largest l_*p*_/*l*_*pran*_ values, which is also clearly illustrated in Fig 10E. As stated in Material and Methods, three regions (9/46d, 9/46v, 46d) in the [45] data correspond to region 46 in our data. Thus, the calculation of the fraction of region 46 in [45] actually involves three targeted regions, thus may have some ambiguity. Besides the three LDC regions 5, 2 and 7b, there are several regions with a large ratio of the wiring cost in the actual network relative to random benchmarks, such as the motor regions 6vb and 4, and the visual regions TPOc and TPOr. In our global network data, these regions have small degrees, and the link recovery by the trade-off model is not strongly significant (Z-score in [− 1.65, 1.65]).

To summarize, the comparison of our global dataset with the more recent, partial dataset of Markov et al. [45] showed that our main results are reliable and robust. Particularly, the connections in our data correspond to strong projections in the new data. The new study revealed that (1) there are even more long-distance connections, which are typically much weaker (Fig 10A and 10B); (2) a few LDCs identified in our dataset of the global network are included in the new dataset (regions 2, 5, 7b, 46), and reassuringly, these four regions also contain most of the long-distance connections in the new dataset (Fig 10C), up to 54.8% of the total weights of long-distance links (x > 30 mm); (3) the total projection cost of these regions (except for region 46 that is represented with some ambiguity) is quite large with respect to random benchmarks in the data of [45] (Fig 10D and 10E). These observations give us confidence that our main finding, of LDC regions violating the cost-efficiency trade-off, likely still holds when the dataset of Markov et al. [45] is expanded to an even more complete connectivity matrix in the future.

## Discussion

This study investigated the intricate connectivity diagram of the primate cortex, particularly basic factors such as wiring cost and processing efficiency that are underlying regional connectivity profiles and the brain’s relationship with advanced functional requirements of segregation and integration. Most (67%) of the connections in the *macaque* monkey cortex can be explained by a trade-off between these two basic but competing factors (Fig 1), higher than the proportion of connections (56%) explained by the rewired network with fixed degrees following the same wiring rules as in the generative model in [14]. The trade-off model also explained the regional connectivity profiles better than the generative model under the same constraint of fixed degrees. Thus, the connectivity of most regions follows the cost-efficiency trade-off. However, a few long-distance connector regions (LDCs) that concentrate a large portion of the long-distance projections are particularly costly in terms of wiring and are poorly explained by the cost-efficiency trade-off (Figs 3–5). Our analysis further provided evidence that the formation of LDCs could be attributed to support advanced functional requirements of the cortical network. First, LDCs (which mainly have intermediate degrees) allow the formation of a hierarchical modular network organization (Fig 7). Second, LDCs are crucial for proper functional segregation (Fig 8A and 8C–8E). Third, LDCs project their connections rather uniformly across different functional modalities (Fig 9A). Fourth, LDCs, forming a dense group overlapping with all functional modalities, allow for efficient communication and integration of the spatially segregated and functionally specialized subsystems (Figs 9B and 8B). Thus LDCs play an important role in the balance of functional segregation and integration, at the expense of larger wiring cost. These observations reflect more deeply a trade-off between physical cost and functional values in the organization of the primate connectome.

### Cost-efficiency trade-off model versus generative models

Recently, research using generative models has made progress in understanding complex brain network features, by employing a few simple spatial embedding and topological connections rules [13, 14, 24–26]. These generative models produce synthetic networks by systematically searching for parameters that explicitly attempt to reproduce multiple statistical features of the real connectome, namely the distribution of clustering, betweenness centrality, edge length, and so on. Generative models showed that projecting connections following a decay of the connectivity probability with distance as observed in the data can recover some statistical properties of real brain networks [24–26]. Some studies also found that the combination of various topological or spatial features well recovers the macroscopic topological properties [14, 24, 47]. Notably, these observed features may be results of basic constraints and organization principles [22]. For example, the topological feature of common neighbors may be rooted in the wiring cost dependence of connections in spatially embedded networks [25]. The connection probability following a distance decay could be the consequence of wiring cost economy, together with the functional requirement of maintaining efficient propagation of signals [14]. Different from approaches using some observed features to recover the other statistical properties of primate brain connectomes, the aim of the present study was to explore the role played by the two fundamental factors of wiring cost and global processing efficiency on the statistical features, the regional connectivity profiles as well as individual links of the *macaque* cortical network. Remarkably, we showed that the cost-efficiency trade-off model that has only one parameter and that does not integrate multiple topological and statistical features into the objective function can also recover these multiple features as well as the optimal generative model (Fig 2), and in addition can much better recover the connectivity matrix (Fig 1). In the cost-efficiency trade-off model, short and long-distance links can be more clearly related to the trade-off between cost and processing efficiency, and the connectivity profiles of most of the regions can be well recovered (Fig 4), except for a few special LDC areas with a particularly large number of long-distance links. The finding of the LDCs revealed new organization features of the cortical connectome and pointed at additional functional requirements of segregation and integration. The generative model, by contrast, did not well recover both short and long-distance links of many areas (Fig 1C and 1D), and it is not as intuitive as for the trade-off model how to associate those uncovered links to functional values. A good recovery across multiple statistical features, regional connectivity profiles and individual connections in the trade-off model provides strong evidence to support the hypothesis of a trade-off of physical cost and functional values in the brain connectome.

Our findings went beyond previous observations on structural and functional constraints on cortical networks. We found that some subsystems, such as the frontal subsystem, are almost fully recovered by the wiring cost minimization only (α = 1), with a recovery rate of 0.98 (Figs 3A and 4A), consistent with previous reports based on component placement optimization [18, 48]). However, several other studies showed that connections in most subsystems are clearly not optimized for wiring cost, and the whole network is not minimally wired [18] when the network topology is fixed in component placement optimization.

Many essential topological properties of brain networks, such as the coexistence of modules and hubs, may be shaped by a trade-off between the wiring cost and processing efficiency when the reconstructed network is only required to have the same total number of links as the real network, as shown in our previous study [27]. Notably, the original generative model [14] with the combination of spatial distance and topological factors was not able to reliably recover the individual degrees (S1A Fig), although it could better recover the degree distribution (S1C Fig) than the cost-efficiency trade-off model [27] (S1D Fig). The trade-off model without fixing the degree [27] can also generate heterogeneous degrees due to the inhomogeneous spatial layout of the areas, and the degrees in the model network are significantly correlated with the degrees in the real network [27] (S1B Fig). However, the correlation value (0.28) was not very high. Thus, these results suggested that the degrees are partially affected by the cost-efficiency trade-off, but it is still likely that the degrees are also affected by other functional requirements. In the present work, we thus fixed the input and output degrees of each node as in the real network, both for the cost-efficiency trade-off model and for the generative model. Preserving the degrees of each region thus already put some effects of the cost-efficiency trade-off on the benchmarks for reconstructing networks. Similar schemes of generating random benchmarks while preserving the node degrees have been widely used in network analysis. When fixing the degrees, some areas with a large degree are forced to have some long-distance connections, which increases the global efficiency and limits the freedom of variation of the network organization. Indeed, the graph distance *L*_*g*_ (the reverse of efficiency) varies much less (1.58~1.74) between efficiency optimal (α = 0) and cost optimal (α = 1) networks for fixed degrees, just half the range (1.62~1.91) compared to the model without fixing degrees [27]. Thus, the cost minimization (α = 1) under fixed degrees already effectively reflects some trade-off between cost and efficiency, and the overall recovery rate of connections is already larger than 60% in the *macaque* cortical network. In the present work, a further trade-off with efficiency refined the cost-efficiency trade-off and further increased the recovery rates. In this case, rewiring a link from short to long distance more strongly affected the wiring cost *L*_*p*_, but only slightly decreased the graph distance *L*_*g*_ (or increase the processing efficiency). Thus, more weight was put on the efficiency to achieve a trade-off, corresponding to an α value close to zero (0.006). However, it is important to stress that cost minimization is still effective here. The efficiency optimal network without the cost constraint (α = 0) had a much larger wiring cost (Fig 1A) and much lower recovery rate (Fig 1B, inset). The further trade-off with the processing efficiency clearly improved the recovery of some long-distance connections in the network, as shown in Fig 1C and S2 Fig, and simultaneously abolished many false-positive links at short distances (Fig 1D).

### The functional role of LDCs is unexplained by the cost-efficiency trade-off model

We further studied the distribution of the long-distance connections on different regions beyond the basic cost-efficiency trade-off and its relationship with functional segregation and integration. Except for regions with low degrees and unreliable statistics, there are only a few regions with intermediate and large degrees, whose connectivity profiles are not well explained by the basic cost-efficiency trade-off model. Our findings elucidated that these regions (LDCs) appear to be crucial for maintaining advanced functional requirements of segregation and integration.

As for functional integration, previous studies have predominantly focused on hubs with the largest number of connections [35, 49]. Recently, the grouping of such high-degree regions as a densely connected core or “rich-club” in the human and non-human primate brains has attracted great attention [11, 32, 50]. Consistent with the previous work, the hubs for the *macaque* cortical network, especially most frontal regions, such as 11, 12o, 12l, 13a, 24, and LIP, form a rich-club and have high diversity coefficient among different functions (Fig 9A). Interestingly, these regions project most connections at short distance, and can be recovered well by the trade-off model (black triangles in Fig 4). Together with our previous findings that hubs are located close to the regional geographical centers, such an organization is wiring economical for the high-degree hubs. Previous study has also revealed that the regions with long-distance connections have a high diversity coefficient among different modules in the mouse brain connectome [26]. Here we showed consistent results in the *macaque* connectome that there are also LDC areas possessing a high diversity coefficient, but these areas mainly have intermediate degrees and are non-hubs structurally, while also forming a strong dense group. These regions appear to be crucial for integration among functional clusters of the *macaque* brain connectome. Indeed, most LDCs correspond to functional hubs, some of which belong to the default mode network. In fMRI studies, functional hubs are detected as regions of a high density of functional connectivity with other regions [11, 36]. Previous studies on the intrinsic activity of the brain identified functional hubs such as the precuneus, posterior and anterior cingulate gyrus, dorsomedial frontal cortex, as well as inferior parietal regions [31, 51, 52]. Among the LDCs that we identified for the Macaque structural network, there are several regions overlapping with the functional hubs in human or *macaque* brain, such as areas 7b (inferior parietal cortex) [4], 23 (posterior cingulate cortex) [53], 46 (DLPFC) [6], and AITd (anterior inferior temporal cortex). Notably, area 23 and 46 are also hubs in the human structure network [30, 54]. Some of the LDC regions, for instance, areas 23, SII and 46, also overlap with the default mode network [55, 56]. The overlapping of some LDCs, consisting mainly of non-hubs and a few hubs in structural connectivity, with functional hubs and the default mode network, suggests that concentration of long-distance connections on LDCs may constitute an anatomical substrate for functional integration, in addition to possessing an intermediate or large number of connections. It would be interesting to extend a similar analysis to human brain to investigate whether there exist long-distance connections concentrated on LDCs that also play important roles in functional performance.

However, counter-intuitively, the formation of LDCs also appears important for proper functional segregation. Recent fMRI studies showed that cognitive functional domains are segregated into different clusters of functional connectivity [4–8, 10–12, 57, 58]. Different from these studies, the current work did not involve functional imaging data for *macaque*. The clustering analysis here is applied to the structural cortical network and compared with the anatomical functional domains distinguished by cytoarchitectonic and myeloarchitectonic features in previous studies [2, 3]. In the future, it will be interesting to compare the structural clusters with functional modules [6, 8]. Our results demonstrated that, under the basic cost-efficiency trade-off, the modeled connectome has a much smaller number of long-distance connections, and only possesses two large functional domains, namely, visual and frontal systems (Fig 7D), mixed with areas from other systems. It appears somewhat counter-intuitive that the real network with many more long-distance connections possesses an intricate segregation of the system into visual, frontal, somatosensory and motor functional subsystems (Fig 8). Here the important point is that the long-distance connections are largely concentrated to LDCs, so that modules can be better preserved by the intra-modular, short-distance connections, and are not strongly mixed by the inter-module long-distance connections. Indeed, if we preserve a similar number of long-distance connections in the real network, but rewire them to avoid high concentration on particular nodes (i.e., abolishing LDCs), the partition of modules (clusters) and the matching with functional divisions is destroyed (R-network, Figs 7 and 8).

### Insights into organization principles and vulnerability of the connectome from cost-efficiency trade-off

Importantly, our findings provide new insights on the organizing principles of primate cortical connectivity. (1) A large number of white matter projections follow the trade-off between the parsimonious requirements of economical wiring and efficient processing, to group the cortical areas into spatially segregated functional domains. (2) To support integrated functional performance, the hubs (e.g., mostly in the frontal system and visual systems) with short-distance connections link the segregated functions of neighboring regions. (3) Furthermore, to integrate the multimodal functions of remote regions, it appears to be necessary to sacrifice the wiring economy of a few regions (i.e., LDCs). Indeed, LDCs concentrate nearly 2/3 of long-distance connections (Fig 4F) and form a dense group (Fig 9C), although most LDCs have average degrees (Fig 4D). In this way, the organization of intermediate-degree LDCs not only shares the load of integrating spatially segregated functions, but also reduces the wiring cost burden of the outstanding hub nodes by absorbing most of the long-distance connections (Fig 6).

Furthermore, concentrating the long-distance connections on LDCs also allows to maintain a proper segregation of the system into intricate subsystems for specialized processing. These new findings provide further support for the hypothesis of a trade-off between physical cost and functional values in the brain network organization.

Importantly, LDCs might be spots of vulnerability in the brain network. Recent studies suggested that functional hubs partially overlap with regions of high metabolic rate and deposition of disease-related agents (such as Amyloid plaque) observed from PET data [31, 36, 59], and are also vulnerable in various neurodegenerative diseases and mental disorders [60–62]. Several studies have started to reveal the importance of long-distance connections for the energy consumption in human functional networks [63]. Other work also indicated relationships between structural hubs and brain disorders or diseases [19]. Intuitively, structural hub regions with a large number of fiber connections may have high metabolic demands, and therefore become vulnerable in disorders and diseases. On the one hand, attaching a large portion of the long-distance links to intermediate-degree LDCs may reduce the risk of energy deficits in hubs. On the other hand, non-hub LDCs, carrying a strong inter-functional communication and integration load based on long-distance connections, might also become metabolically highly demanding, and may also be vulnerable to disruptions in energy supply and other attacks. Indeed, some LDCs correspond to vulnerable regions, such as areas 23 and 46, in chronic progressive neurodegenerative diseases [36, 64]. Our work, thus, provides a fresh perspective for investigating the relationship between multiple constraints and disease vulnerability in the structural and functional networks of the human brain.

### Reliability and robustness of the findings

In the current work, our results are subject to some inherent limitations imposed by the experimental data. For example, recent data [45] provided weighted *macaque* cortical connectivity, which we used to test the reliability of our findings (Fig 10). However, the weighted dataset, while more detailed than previous data collations, is far from complete. Thus, it will be promising to re-explore basic factors on structural connectivity once a more complete weighted connectome is available.

Presently, there may be concern that incompleteness of the CoCoMac database may contribute to our finding that most of the LDCs are not high-degree hubs of the network. The Markov et al. data [37] showed that many brain regions are connected by very weak fiber projections, leading to high density of binary connectivity at the area level, which might imply that there are not really any outstanding hubs. Measuring degrees by binary connectivity, that is, regarding many very weak links as equally important as the strong ones appears not very appropriate, given that the weight values span orders of magnitude. Consequently, the total weight of a node may be a more reasonable measure of degree (appropriately reflecting connectivity at the level of the relative fiber density), and the total weights are mainly contributed by the strong links. Indeed, the total input weight of the areas covered so far is quite heterogeneous. Thus, it is likely that the network still has hubs in terms of total node weight, if the Markov et al. data are extended to provide complete cortical coverage.

Our detailed comparison between the two data sets (Fig 10) showed that our primary dataset, which was collated and further developed from CoCoMac, is reliable and captures the majority of the significant and strong projections for the corresponding areas covered in the Markov et al. data. Therefore, measuring the binary degree in our un-weighted global network appears as a reasonable estimation of the total weights of a node. Our observation, that four of the LDCs identified in our dataset also concentrate the majority of the long-distance projection weights in the Markov et al. data, suggested that the LDCs would remain as LDCs if the new data set would be completed. It is also not very likely that these areas will be drastically changed to hubs in terms of total weights, which could happen only if many and a large portion of strong links were missed for these areas in the CoCoMac data. Therefore, it will be interesting to test if LDCs can indeed be identified as non-hubs of the structural connectivity once a more complete weighted connectome becomes available.

### Limitations and outlook

The cost-efficiency trade-off model with fixed degrees can recover most of the connections of the whole *macaque* cortical network, which allows to reveal new organization features by further analysis of a few regions with low recovery rates by the model. However, fixing degrees limits the capacity of the model to explore the mechanisms underlying important features in the degree sequences, such as degree distribution, hubs and degree correlations. Further studies of the cost-function trade-off may need to develop more sophisticated quantification of the functional values related to degrees in order to better elucidate the underlying mechanisms.

While the global efficiency of the interregional cortical network may partially capture the basic functionality of the brain at the systems level by using the strongly simplified assumption of identical network nodes, it should be stressed that actual brain functions are implemented by highly specialized cortical areas, comprising heterogeneous local circuits and displaying complex dynamical activity. Interestingly, these two levels of organization are also interrelated. For example, similarity in regional neuronal density is closely related to the probability of interregional connection [65–68], and the regional synaptic spine density (and consequently the response time of the local circuit) is related to a gradient of cortical areas [69] (which is also roughly related to the total degrees of areas). Therefore, most likely the basic principle of a trade-off between physical cost and functional values could be operating to shape the network structure and dynamics across different levels. Our own recent work [70] showed that the co-organization of salient multi-scale dynamical features as typically observed in electrophysiological experiments, including irregular firing of individual neurons, clustered firing of neuron groups in the form of critical avalanches and the emergence of stochastic oscillations of the population, indeed reflects a cost-efficient neuronal information capacity with economical firing rates. In the future, it will be important to study cost-efficiency trade-offs in an integrated manner in terms of both neuronal connectivity and activity and in specific neuronal information processing tasks across multiple levels of brain organization.

### Conclusion

Our study suggests that primate anatomical connectivity, comprising characteristic topological features as well as specific regional connectivity profiles and individual connections, is shaped by a basic cost-efficiency trade-off as well as advanced functional requirements, reflected by a special group of long-distance connector regions that are crucial for functional segregation and integration. Together, these findings support the hypothesis of a trade-off between physical cost and functional values in brain network organization [13]. Our work, moreover, illuminates the potential inherent vulnerability of the cortical connectome as a result of the competition between energy cost and functional values, which were not identified by previous topological analyses of cortical connectivity.

## Materials and methods

### *Macaque* cortico-cortical network

We analyzed the connectivity of the *macaque* cortical network and its relationship with the three-dimensional spatial layout of the network components and compared the original network to various reconstructed networks in order to understand the impact of multiple structuring factors. The analyzed *macaque* connectivity data was based on anatomical tract-tracing and adapted from a dataset of 94 cortical regions and 2,390 directed projections among them [18]. The connectivity data and three-dimensional spatial positions (the average surface coordinate) of each cortical region were obtained from http://www.biological-networks.org. However, the dataset did not provide complete coverage of cortical regions. Especially the divisions of motor regions were quite coarse, with incomplete connection data of several regions (e.g., motor regions 4 and 6 which cover a large territory, 6.5% of neocortex). In a previous study [27], we improved and expanded the dataset to 103 regions using a more detailed parcellation of the motor regions based on the CoCoMac database [43]. The spatial positions of the newly added regions were taken as the average surface 3-D coordinate estimated from surface parcellation using the CARET software (http://sumsdb.wustl.edu/sums/index.jsp). Consequently, the improved dataset was also used in the present study. The cortical network of the nonhuman primate (*macaque* monkey) studied here has N = 103 regions and K = 2518 connections in total [27]. The labels of the regions are listed in S1 Table of SI.

This network was also compared to a recent systematic tract-tracer study [45, 71, 72], for which, however, only an incomplete square matrix (for only 29 out of 91 candidate regions) is currently available; see details in the final section of Results.

### Connectivity optimization and reconstructed networks

We reconstructed the cortical network connections based on a variety of objective functions, while preserving the spatial positions as well as both the input and output degree of the regions as in the real cortical network. Network connectivity can be described as a matrix {*A*_*ij*_} with *A*_*ij*_ = 1 if there is a link from region *j* to *i*, and *A*_*ij*_ = 0 otherwise. The reconstructed networks were obtained by minimizing an objective function combining the wiring cost and processing efficiency,

$$L=\left(1-\alpha \right)L_{g}+\alpha L_{p},$$ (1)

where α is a parameter to represent the relative weight of the normalized physical length $L_{p}=l_{p}/l_{p}^{max}$ which reflects the influence of the wiring cost, and the normalized graph length $L_{g}=l_{g}/l_{g}^{max}$, representing the influence of the processing efficiency. Here *l*_*p*_ is the total wiring length of the links and *l*_*g*_ is the sum of the shortest path lengths between all pairs of nodes in the network. $l_{p}^{max}$ is obtained at α = 0 when minimizing *l*_*g*_ without considering the wiring cost, and $l_{g}^{max}$ is obtained at α = 1 when minimizing *l*_*p*_ without considering the efficiency.

In the simulation, we computed *l*_*g*_ as the reciprocal value of the global network efficiency, *l*_*g*_ = 1/*e*_*g*_, where *e*_*g*_ is defined as $e_{g}=\frac{1}{N\left(N-1\right)}\Sigma_{i\neq j\in G}\frac{1}{l_{ij}}$ where *l*_*ij*_ is the shortest pathlength between the nodes *i* and *j* [73]. In this way, we avoided the numerical problem of isolated nodes (where some path lengths would be ∞). Disconnection of nodes can be naturally avoided in the optimization processes for α < 1, because disconnection leads to large *l*_*g*_ The fiber length between the regions was estimated by Euclidean distance between the spatial positions of the regions. Euclidean distance is inexact, because the fiber tracts do not strictly follow the straightest trajectory. However, based on the linear proportional relationship between the fiber length and Euclidean distance within a hemisphere [74], the Euclidean distance is a good approximation of fiber length, and has been widely applied for the primate brain [49, 75, 76]. The wiring length *l*_*p*_ is taken as the sum of the distances between connected areas.

We applied a simulated annealing optimization algorithm [77] to search for network configurations that minimize the objective function *L*. The algorithm was implemented as follows: starting with a random network and a high temperature *T*_*0*_, the temperature was reduced as*T*_*n*+1_ = *T*_*n*_/*n*. At each temperature level, the network was rewired for 1000 steps by exchanging the connections of two pairs of randomly selected notes (disconnected networks were discarded). If *L* after switching was smaller than before switching, i.e., Δ*L<0*, the switching was accepted; otherwise, the operation of switching was accepted with a probability exp(−Δ*L*/*T*). The program was terminated whenΔ*L* ≤ 10^−5^ [27].

Reconstructed connectivity $\left\{\bar{A_{ij}}\right\}$ was obtained for each α from 50 realizations of the optimized networks from different initial random networks. The probability for finding a link is *P*_*ij*_ = *N*_*ij*_/50, where *N*_*ij*_ is the number of realizations with a link from area *j* to *i*. The reconstructed connectivity is $\left\{\bar{A_{ij}}\right\}=1$ if *P*_*ij*_ ≥ *P*_*T*_ and A_*ij*_ = 0 otherwise, where the threshold *P*_*T*_ is set for a given α such that the total number of links of the reconstructed network is the same as the real network *K* = 2518 (in fact, the closest possible value to *K*, due to discreteness of *P*_*ij*_ from 50 realizations). The thresholds differ slightly at different α, but all are larger than 0.5, which means that the corresponding link is appearing in more than 50% of realizations, showing good consistency of the optimization algorithm.

### Generative model

In this part of the study, we compared the performance of recovering different statistical features, regional connectivity profiles as well as individual connections of the real *macaque* connectome by the cost-efficiency trade-off model and a recently proposed generative model [14]. This generative model aims to generate model networks by combing spatial and topological rules, and searches for parameters that can best reproduce multiple statistical features of the real network. Starting with a sparse seed network (464 edges among 16 regions, both randomly selected, about 15% of the nodes and links of the real network [14]), edges were added one at each time over a series of steps until the remaining M = 2518–464 = 2054 total connections were added. At each step, the unconnected nodes, u and v, were connected with a probability P(u, v), which is given by:

$$P\left(u,v\right)=\;E{\left(u,v\right)}^{\eta }\times K{\left(u,v\right)}^{\gamma },$$ (2)

where E(u,v) denotes the Euclidean distance between brain regions u and v, and K(u,v) represents a non-geometric relationship between nodes u and v, which contains 12 different generative models as listed in S3 Table. We applied the matlab function “generative model.m” of the Brain Connectivity toolbox (https://www.nitrc.org/projects/bct/) to generate the synthetic networks at different control parameters *η* and *γ*. The optimal generative model can closely recover the degree distribution (S1C Fig), but it cannot reliably reproduce the degree sequence, since the (total) degree of each node from the model is not significantly correlated with that of the real network (S1A Fig). On the other hand, although the cost-efficiency model without fixing the degrees does not recover the degree distribution as well as the generative model, it could slightly better recover the degrees of nodes, and the correlation between the degree of model and the real network becomes significant (S1B and S1D Fig).

To have a fair comparison of the generative model with the cost-efficiency model under fixed degrees, we extended the generative model to have the constraint of fixed degrees as in the real network. The model network is obtained by rewiring initial random network while fixing degrees to approach the wiring probability as that in the generative model (Eq 2). Starting with a random network with the degrees fixed as that in the real *macaque* brain network, randomly pick two (directed) links connecting two pairs of regions (*u*_1_, *v*_1_ and *u*_2_, *v*_2_), given that there are no crossing-connections between the two groups, i.e., *A*(*u*_1_, *v*_1_) = 1, *A*(*u*_2_, *v*_2_) = 1, *A*(*u*_1_, *v*_2_) = 0, and *A*(*u*_2_, *v*_1_) = 0. Then with a probability *P*(*u*_1_, *v*_2_) * *P*(*u*_2_, *v*_1_), we exchanged the connections for these two pairs of region, namely *A*(*u*_1_, *v*1) = 0, *A*(*u*_2_, *v*_2_) = 0, *A*(*u*_1_, *v*_2_) = 1, and *A*(*u*_2_, *v*_1_) = 1. Here

$$P\left(u_{1},v_{2}\right)*P\left(u_{2},v_{1}\right)=\;E{\left(u_{1},v_{2}\right)}^{\eta }\times K{\left(u_{1},v_{2}\right)}^{\gamma }*E{\left(u_{2},v_{1}\right)}^{\eta }\times K{\left(u_{2},v_{1}\right)}^{\gamma }$$ (3)

is the probability to place simultaneously two independent links following the generative rules (Eq 2) as described above. The rewiring will repeat for large enough time steps until 200,000 pairs rewired. So the network is supposed to follow the generative rules, but maintaining the input and output degrees as in the real network.*K*(*u*, *v*) reflecting topological relationship also contains 12 different generative models as listed in S3 Table. We generated the rewired networks at different control parameters *η* and *γ* as in the case without fixing the degrees.

### Evaluating fitness in the generated networks for different features

In the previous work [14], the optimized generative network was obtained by searching the parameter space (*η*, *γ*) to achieve the lowest energy, which quantifies the similarity of the generated network to different features in the real network. The energy of the generated network was defined as:

$$\text{E}=\text{max}(KS_{degree},KS_{clustering},KS_{betweenness},KS_{edgelength}),$$

where KS is the Kolmogorov-Smirnov statistics quantifying the discrepancy between the synthetic and the real *macaque* cortical network in terms of their statistical distribution of degree, clustering, betweenness centrality, and edge length. For the extended generative model with fixed degrees as in the real network, *KS*_*degree*_ = 0, which does not affect the definition of energy as the upper bound of the measures. Clustering measures the fraction of a node’s neighbors that are connected to each other. Betweenness centrality of a node is the number of the shortest paths in the network that pass through the given node. Edge length refers to the Euclidean distance between two regions of the connection. Thus, the optimization process searches for the network configuration under the generative rules that maximizes the similarity to multiple statistical features of the real network. When applied to the *macaque* connectome, the generative model with the lowest energy is obtained when the spatial distance is combined with the matching index (i.e., K(u, v), which is the ratio of common neighbors to total neighbors of two nodes u and v), as is consistent with a previous study of the human connectome [14] (Fig 1B). The different KS measures were also applied to the reconstructed network from the cost-efficiency trade-off model, and compared with KS for the optimized synthetic network from the generative model (Fig 2).

### Comparing reconstructed and real networks matrices and computing recovery rates

The adjacency matrices $\bar{A}$ of the reconstructed networks (from both generative model and cost-efficiency trade-off model) were compared to *A* of the real network in different ways. (1) We counted the number of overlapping entries between $\bar{A}$ and *A*, obtaining *K*_*r*1_ and *K*_*r*0_ respectively for the connected pairs (*A*_*ij*_ = 1) and unconnected pairs (*A*_*ij*_ = 0) in the real network. The corresponding recovery rates were *r*_1_ = *K*_*r*1_/*K*_1_ and *r*_0_ = *K*_*r*0_/*K*_0_ for the *K*_1_ = *K* entries *A*_*ij*_ = 1 and *K*_0_ = *N*(*N* − 1) − *K* non-diagonal entries *A*_*ij*_ = 0. The values *r*_1_ and *r*_0_ were also obtained, to measure the recovery rates for pairs of areas separated within a range of distances (Fig 1B and 1C), and to quantify the recovery of connectivity of each area in the network (Fig 4). Recovery rates from the reconstructed networks were compared to random benchmarks where the number of input and output links of each area was preserved as in the real network, but the connections were randomly rewired by exchanging links of two pairs of randomly selected areas.

### Rewiring long-distance links to obtain the R-network

As detailed in the Results, there are about 33% of connections in the real networks which cannot be recovered by the best reconstructed network from the cost-efficiency trade-off model. The unrecovered connections are mainly long-distant links and are concentrated on a few special areas termed long distance connectors (LDCs). To study the impact of the concentration of links on LDCs, we randomly rewired the unrecovered links of LDCs to obtain an ‘R-network’ while preserving the distribution of physical distances as in the real network. In this way, only the concentration of long-distance links on LDCs was destroyed while the wiring cost remained the same. Thus, comparing the real network with R-networks, we could explore the functional influence of LDCs while excluding the contribution of the wiring cost. The R-network had similar efficiency (*l*_*g*_ = 1.81) as the real network (*l*_*greal*_ = 1.85), but its long-distance connections were not concentrated on the LDCs.

### Hierarchical tree of network from connectivity matrix

Applying established modular division methods [78], we found that there are only two modules in the real *macaque* cortical network [27], roughly separating the visual and frontal cortex, both mixed with areas from other functional systems. The reason, why the traditional modular division does not well capture the functional segregation, may be because both the size and the intra-connection density for different functional subsystems are quite heterogeneous. To better illustrate and detect the relationship between the clustering of anatomical connectivity and functional segregation, we studied the clustering of the brain cortical areas by analyzing the hierarchical tree of the connectivity matrix. The hierarchical tree (Fig 7) was obtained using the similarity measure {*S*_*ij*_} computed from the connectivity matrix *A*, *S*_*ij*_ = *M*_*ij*_/(*K*_*i*_ *+ K*_*j*_ − *M*_*ij*_), namely, the ratio of common neighbors *M*_*ij*_ over the total distinct neighbors of two nodes *i* and *j*. The MATLAB function pdist was used to obtain the hierarchical tree (dendrogram) using the dissimilarity 1 − *S*_*ij*_. The connectivity modules from the sub-trees cut out at different thresholds (see below) were compared to functional subdivisions of the network as described in the next section. The limitation of the traditional modular division not well capturing the functional segregation is due to the fact that a uniform threshold is chosen to maximize the modularity, which however mixes different trees.

### Functional segregation in three types of networks

To calculate the degree of matching between the hierarchical trees and brain functions, we first identified sub-trees dominated by a certain function. We cut the hierarchical tree from the top, with the threshold varying from 1 to 0, to obtain different sub-trees dominated by the corresponding functions. At each threshold, we calculated for each sub-tree the fraction of regions from different functional subsystem with respect to the total number of regions in the tree. Once the fraction for some functional subsystem in specific sub-tree was larger than 0.5, this sub-tree was retained and not divided further. We cut the hierarchical trees until we found all sub-trees with the fraction of regions from some functional subsystem to all regions in the sub-tree larger than 0.5 and the number of regions from a dominating function larger than 3, since further partition would make too many fragmental and small trees.

We illustrate the method by detecting the subtrees of the hierarchical tree in the real network in Fig 8A. At threshold value 0.67, the first sub-tree (Tree 1) was obtained containing only motor areas (matching rate 100%). The other large branch at this threshold at the right part of Fig 8A contains cortical regions from different functional systems and is not dominated by one of the functions. Then we proceeded to cut this branch further into two major branches at a lower threshold of 0.65 and obtained the second sub-tree (Tree 2) containing only visual regions (matching rate 100%). Now the remaining middle branch still contains regions from different functions, and the process continued until we obtained all sub-trees where the number of regions from a dominating function was larger than 3. The remaining regions that were not included in the trees identified above were put into a group called”non-clusters”. In this way, a total of 6 sub-trees were obtained for the real network, as indicated in Fig 8A, and they are graphically presented in Fig 8B of the main text.

The ratio of dominance for a certain function in each of the hierarchical trees in the real network is very high, 100% for 5 trees and 86% for tree 4 (with just 1 region mismatched).

### Functional diversity coefficient C_*i*_ of cortical regions

For each region, we calculated how the links were distributed among the five functional systems (visual, somatosensory, motor, temporal and frontal), $p_{i}\left(\text{J}\right)=\frac{k_{i,J}}{k_{i}}$, where *k*_*i*_ is the total degree of region *i* and *k*_*i*,*J*_ the number of connections linking region *i* with regions of functional system *J*. The functional diversity coefficient of a region can be indexed by the entropy of the link distribution as: $C_{i}=-\sum_{J=1}^{5}p_{i}\left(J\right)\text{ln}\left[p_{i}\left(J\right)\right]$, as applied in [79]. If node *i* ∈ *J* has only internal links within a functional system *J*, then *P*_*i*_ (*J*) = 1 and *P*_*i*_ (*J*′) = 0 for any other *J*′≠ *J*, hence the functional diversity coefficient *C*_*i*_ = 0. For the opposite case, if the links of a region *i* are uniformly distributed in 5 different function regions, then *C*_*i*_ achieves the maximum value ln 5 = 1.61.

### Dense groups in the network

A set of nodes in the network is said to form a dense group when the connection density θ among the nodes is significantly larger (by above one standard deviation) than the expected connection density due to the nodes′ degrees in randomized networks. The calculation of the density is exactly the same as done for rich-clubs of the degree-rich subsets. Such dense groups formed by degree-rich hubs are called rich-clubs [32]. In this study, we assessed the properties for the groups formed by LDCs, which mainly are non-hubs. The connection density θ among LDCs is significantly higher than for random benchmarks (Fig 9B), thus they form a “dense group”.

### Testing the reliability of findings in an additional dataset

The primate cortical network used in this study has 103 regions and 2518 connections in total. However, such a dataset established from the collation of several individual studies available in the literature may have some limitations; for example, the connections of some cortical regions may not be as well characterized as others. Thus, it is important to verify the reliability of the data with reference to other datasets and test the robustness of our findings.

Specifically, the group of Kennedy has recently undertaken a systematic effort to gather cortical connectivity data for the *macaque* monkey using retrograde tracer injections, resulting in a series of papers [30, 45, 71, 72] about the partial network of 1615 connections formed by afferent (input) links to a subset of 29 targeted regions from a total of 91 cortical regions. These targeted regions are sampled from different functional systems. This dataset provides the weight index (FLNe index) for region-to-region connections, which represents the fraction of labeled neurons in a source region relative to the total number of labeled neurons from all possible source regions extrinsic to a targeted region.

However, there are two major limitations and challenges in directly employing this dataset in the present cost-efficiency trade-off study. Firstly and most importantly, the dataset is still far from being complete (providing only about 1/3 of coverage of the whole cortex, and only the input connections are complete for the covered areas); thus, is not representative for applying the cost-effciency trade-off model which considers the wiring cost and processing efficiency of the global network. Secondly, the weight index provides the relative strength of the input of a targeted region from different sources, and the efficiency measure and the rewiring procedure would need to consider the projections weights. Note that the projection weights in *macaque* have a very broad range, spanning 5-order of magnitudes [37]. While very weak links between areas could still be functionally useful, at least at the neuronal level, a simple linear measure of the contribution of the connection weights to the efficiency would not be able to capture its subtle functionality. This issue needs further exploration in the future.

Our detailed comparison between the two data sets (Results, Fig 10) showed that our dataset collated and developed from CoCoMac is reliable when compared to the Markov et al. dataset [80], since it captures the significant and strong projections of the corresponding areas covered in the Markov et al. dataset. Therefore, the CoCoMac data, providing relatively complete cortical coverage, but incomplete characterization of some of the moderately or very weak links, are more suitable at this stage for applying the cost-efficiency trade-off model.

While the dataset is expected to become more complete in the future and an improved cost-efficiency trade-off analysis including the weighted information could then be developed, the current partial information may still be useful for some comparison with our dataset to provide an indication of the reliability and robustness of our findings.

The parcellation of the data by [45] was based on the Felleman and van Essen (1991) atlas. The present data is also based on the Felleman and van Essen (1991) atlas, combined with the Lewis and van Essen (2000) atlas for a more detailed parcellation of the motor system [42]. By comparing the different atlases in CARET, we found that the 91 regions [45] correspond to 74 regions in our data. The 29 targeted regions correspond to 24 regions in our dataset, while the regions STPr, ProM, 10 have no correspondence in our data, and regions 9/46d, 9/46v, 46d all correspond to region 46 in our dataset. We carried out more detailed analyses of the common sub-matrices of 74 × 24 nodes in both datasets. The links of our data in the 74 × 24 sub-matrix highly overlap (78.6%) with those by [45]. For this sub-matrix, only few links in our data do not appear in [45].

### Calculating the fraction of wiring cost of regions in the real cortical network with respect to that in random weighted networks

In this study, we evaluated the wiring cost in the real network by comparing it to that of the random network with the same input and output degrees as in the real network. The fraction of wiring cost with respect to random benchmarks was calculated by l_*p*_/*l*_*pran*_ both in 24 regions of data by [45] (afferents from 74 regions) and our global network data (afferents from 103 regions) for comparison. Importantly, since the data by [45] contain the afferent projections to the 24 regions, we only consider the afferent direction in calculating l_*p*_/*l*_*pran*_ for both datasets. For the weighted links, we can obtain the projection cost (weighted distance) for each targeted region *i* as $l_{p}^{i}=\sum_{j}w_{ij}*A_{ij}*d_{ij}$, where *A*_*ij*_ = 0 or 1 represents the existence of a projection to region *i* from region *j*, *w*_*ij*_ is the weight and *d*_*ij*_ is the Euclidean distance between region *i* and *j*. To obtain corresponding random networks, the weighted link *w*_*ij*_ was randomly shuffled for the index *j* among the 74 cortical regions and the cost $l_{p_{ran}}^{i}$ calculated accordingly. For unweighted links in our data, *w*_*ij*_ = 1 for all the links.

## Supporting information

S1 Table The adjacent matrix of the *macaque* cortical network. The element of this matrix *A*_*ij*_ = 1 represents the connectivity from region *j* to region *i*.
(TXT)

S2 Table The list of the 3-D coordinates of 103 cortical areas in *macaque*. (TXT)

S3 Table List of 12 generative models. (XLSX)

S1 Fig Recovery of degree sequence and degree distribution by the original generative model [14] without fixing the degrees (left panel, A and C) and by the cost-efficiency trade-off model without fixing the degrees [27] (right panel, B and D). (A, B) The (total) degrees of each node in the model vs. that in the real *macaque* cortical network with 95% confidence interval (the dash lines). (C, D) Comparison of (cumulative) degree distribution between the model (red line) and the real *macaque* brain network (black line).
(EPS)

S2 Fig Recovery rate of the trade-off model in short and long distance ranges. (A) Recovery rate *r*_0_ for the unconnected pairs with short distance (x < 30 mm) in the reconstructed networks as a function of α. (B) Recovery rate *r*_1_ for the connections with long distance (x > 30 mm) as a function of α.
(TIF)

S3 FigRecovered and unrecovered links, complementary to Fig 3A and 3B in the main text.(A) The adjacent matrix $\bar{A}$ of the reconstructed network at α = 0.006. The red color represents the recovered links and the green color denotes links that are unrecovered but redistributed in the reconstructed network at α = 0.006. (B) Schematics of the re-distribution of the unrecovered real links in the reconstructed network (green arrows). The width of the arrows indicates the number of connections (roughly). Typically, the original long-range connections were rewired to spatial neighborhood. The cortical surface is constructed by the CARET software (http://sumsdb.wustl.edu/sums/index.jsp).(EPS)

S4 Fig The recovery for the regional connectivity profile by the optimal generative model with fixed degrees. (A) Recovery rate *R*_recov_ for each area. (B) The Z-score of the recovery rate Z_*R*_(*i*) of the synthetic network from the generative model when compared to the random benchmark networks. (C) The Z-score of the areas sorted by the rank of total degree in the real network. The vertical dashed lines in (A) and (B) indicate the separation of the functional systems (visual (V): red; somatosensory (S): green; motor (M): blue; temporal (T): gray and frontal (F): black). The horizontal dashed lines in (B) and (C) indicate the range of Z-score in [− 1.65, 1.65]. The vertical dashed line in (C) separates the areas to the left with small degrees.
(TIF)

S5 Fig The number of special cortical areas whose connections cannot be well recovered by the trade-off model at different α. (A) The number of areas with Z-score Z(*i*) < − 1.65. (B) The number of areas with Z_*R*_(*i*) < 1.65.
(EPS)

S6 Fig (A) The mean distance (black bar) and (B) the mean connectivity density (white bar) for areas within the same sub-tree and between different sub-trees in the real networks. The error-bars in (A) represent the standard deviations of the physical distance in the ensemble of all links within sub-trees.
(EPS)
