# Supplementary figures and images for: Features of spatial and functional segregation and integration of the primate connectome revealed by trade-off between wiring cost and efficiency
Source: PLoS Comput Biol. 2017 Sep 29;13(9):e1005776. doi: 10.1371/journal.pcbi.1005776 (PMC5645157; doi:10.1371/journal.pcbi.1005776)

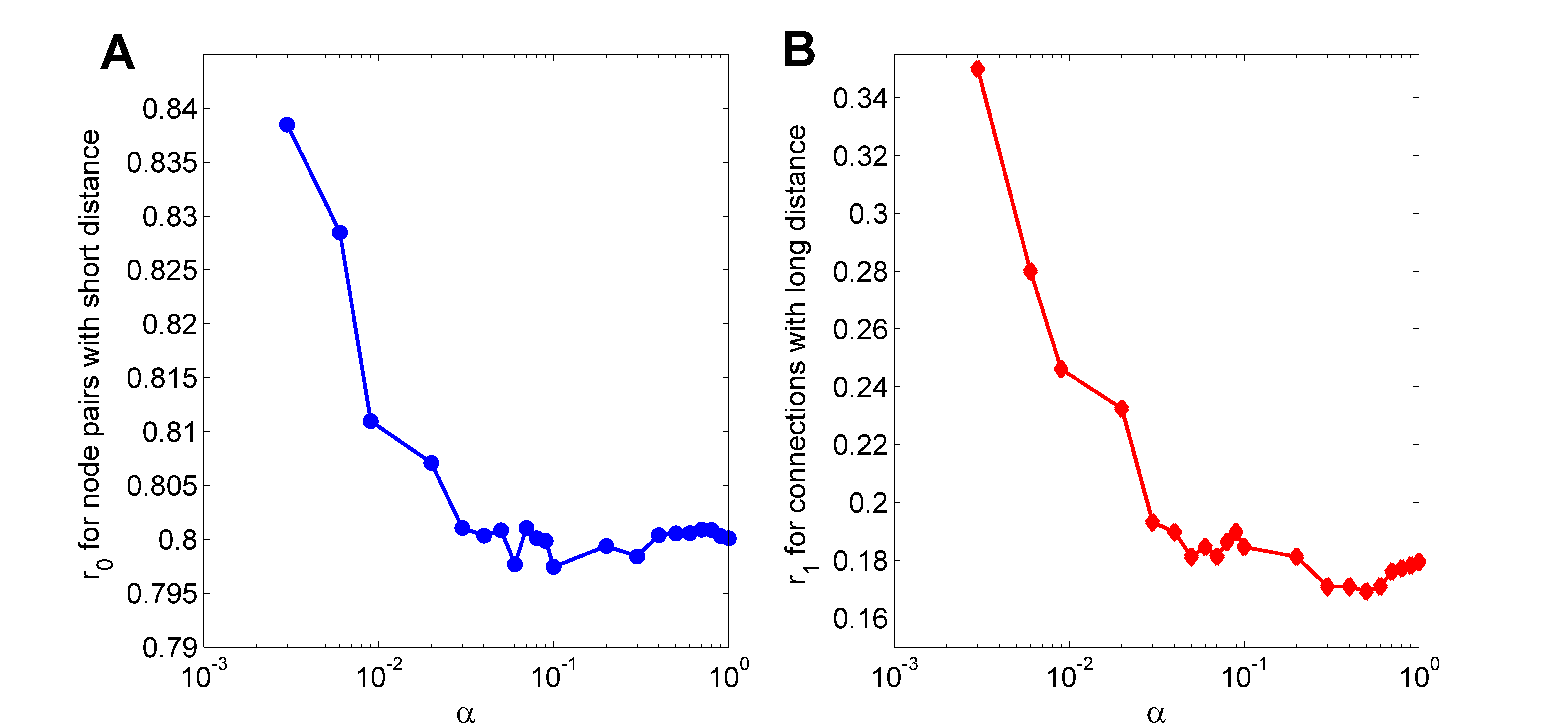

Supplement: S2 Fig — (A) Recovery rate r0 for the unconnected pairs with short distance (x < 30 mm) in the reconstructed networks as a function of α. (B) Recovery rate r1 for the connections with long distance (x > 30 mm) as a function of α. (TIF) [file pcbi.1005776.s005.tif]

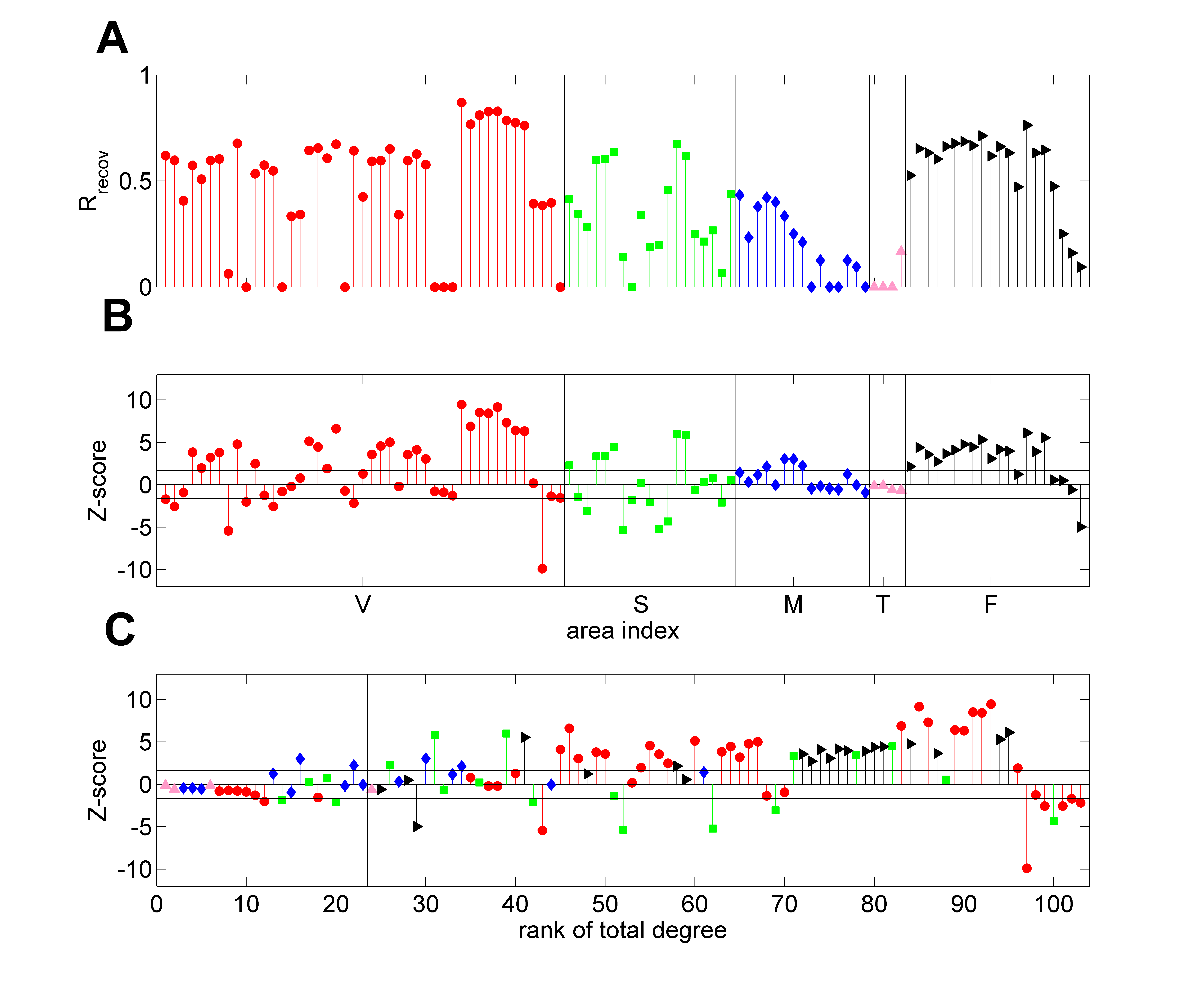

Supplement: S4 Fig — (A) Recovery rate Rrecov for each area. (B) The Z-score of the recovery rate ZR(i) of the synthetic network from the generative model when compared to the random benchmark networks. (C) The Z-score of the areas sorted by the rank of total degree in the real network. The vertical dashed lines in (A) and (B) indicate the separation of the functional systems (visual (V): red; somatosensory (S): green; motor (M): blue; temporal (T): gray and frontal (F): black). The horizontal dashed lines in (B) and (C) indicate the range of Z-score in [− 1.65, 1.65]. The vertical dashed line in (C) separates the areas to the left with small degrees. (TIF) [file pcbi.1005776.s007.tif]
